# Evidence of an oceanic impact and megatsunami sedimentation in Chryse Planitia, Mars

**DOI:** 10.1038/s41598-022-18082-2

**Published:** 2022-12-01

**Authors:** J. Alexis P. Rodriguez, Darrel K. Robertson, Jeffrey S. Kargel, Victor R. Baker, Daniel C. Berman, Jacob Cohen, Francois Costard, Goro Komatsu, Anthony Lopez, Hideaki Miyamoto, Mario Zarroca

**Affiliations:** 1 Planetary Science Institute, 1700 East Fort Lowell Road, Suite 106, Tucson, AZ 85719-2395 USA; 2 NASA Ames Research Center, Moffett Field, CA 94035 USA; 3 Department of Hydrology and Atmospheric Sciences, University of Arizona, Tucson, AZ 85721 USA; 4 GEOPS-Géosciences Paris Sud, Université Paris-Sud, CNRS, Université Paris-Saclay, 91405 Orsay, France; 5 International Research School of Planetary Sciences, Università D’Annunzio, Viale Pindaro 42, 65127 Pescara, Italy; 6 Department of Systems Innovation, University of Tokyo, Tokyo, 113-8656 Japan; 7 External Geodynamics and Hydrogeology Group, Department of Geology, Autonomous University of Barcelona, 08193 Bellaterra, Barcelona, Spain

**Keywords:** Planetary science, Geomorphology

## Abstract

In 1976, NASA's Viking 1 Lander (V1L) was the first spacecraft to operate successfully on the Martian surface. The V1L landed near the terminus of an enormous catastrophic flood channel, Maja Valles. However, instead of the expected megaflood record, its cameras imaged a boulder-strewn surface of elusive origin. We identified a 110-km-diameter impact crater (Pohl) ~ 900 km northeast of the landing site, stratigraphically positioned (a) above catastrophic flood-eroded surfaces formed ~ 3.4 Ga during a period of northern plains oceanic inundation and (b) below the younger of two previously hypothesized megatsunami deposits. These stratigraphic relationships suggest that a marine impact likely formed the crater. Our simulated impact-generated megatsunami run-ups closely match the mapped older megatsunami deposit's margins and predict fronts reaching the V1L site. The site's location along a highland-facing lobe aligned to erosional grooves supports a megatsunami origin. Our mapping also shows that Pohl's knobby rim regionally represents a broader history of megatsunami modification involving circum-oceanic glaciation and sedimentary extrusions extending beyond the recorded megatsunami emplacement in Chryse Planitia. Our findings allow that rocks and soil salts at the landing site are of marine origin, inviting the scientific reconsideration of information gathered from the first in-situ measurements on Mars.

## Introduction

**Figure 1:**
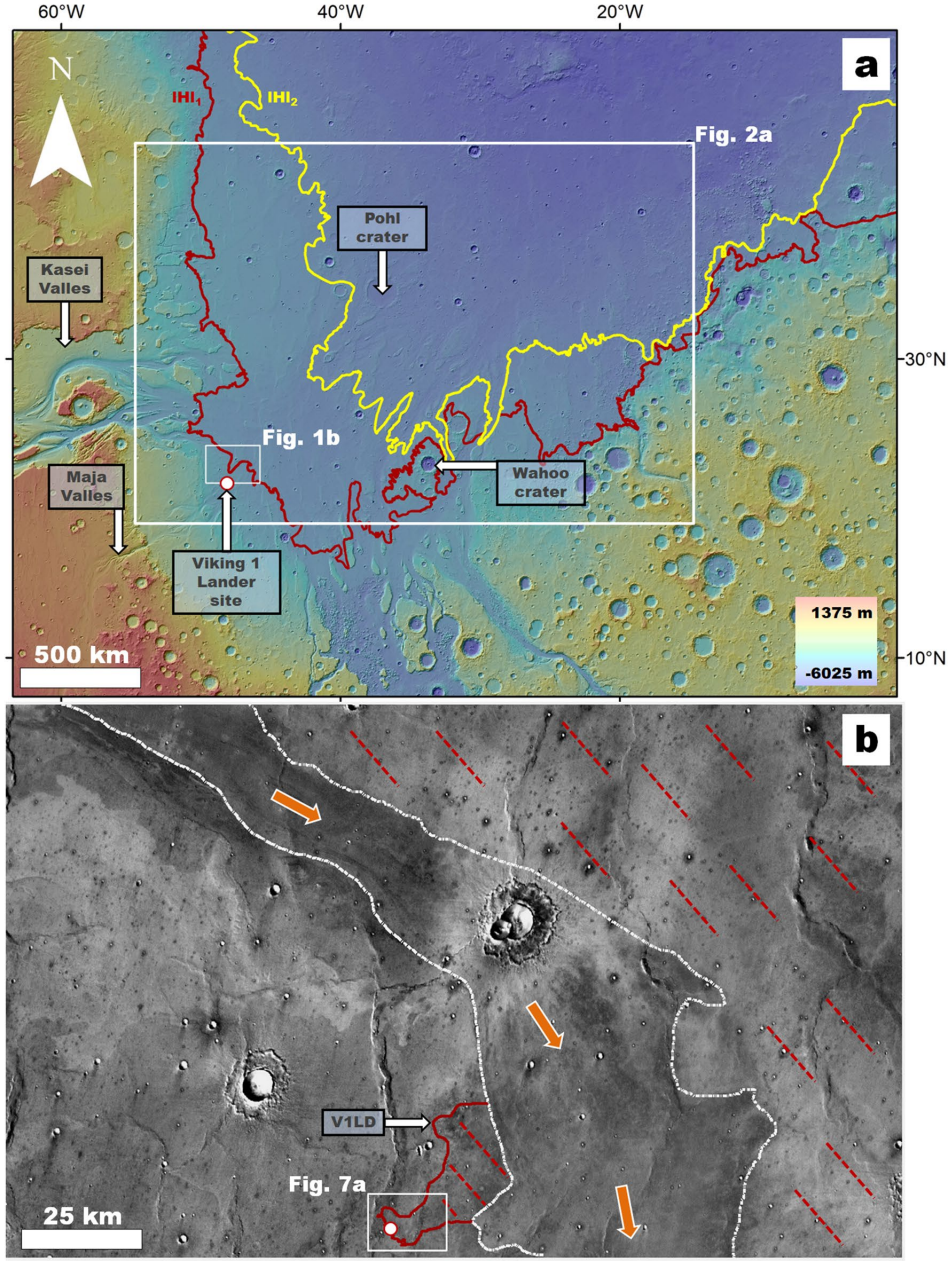
**(a)** View of the Chryse Planitia region showing the margins of older and younger megatsunamis (i.e., unit members lHl_1_[solid red line] and lHl_2_ [solid yellow line] as mapped by Rodriguez, et al.^6^) and the location of Pohl crater, Wahoo crater, Maja Valles, and Kasei Valles. The red-circled white dot indicates the location of the Viking 1 Lander (V1L). The map base is a MOLA DEM overlying a shaded relief (460 m per pixel, credit: MOLA Science Team, MSS, JPL, NASA). **(b)** Close-up view on panel (**a**) showing the revised extent of the older megatsunami (frontal margins [solid red line], covered areas [dashed red lines]). The white dotted lines indicate the margins of a Kasei Valles channel dissecting the deposit. The red-circled white dot marks the location of the V1L. The orange arrows reconstruct the overall flow direction from Kasei Valles (Figs. S4 and S5). The base image is part of a THEMIS Day IR (infrared) Global Mosaic (http://www.mars.asu.edu/data/, 100 m per pixel, credit: Christensen, et al.^7^). We produced this figure using Esri's ArcGIS 10.3 (http://www.esri.com/software/arcgis).

NASA's 1971 Mariner 9 spacecraft discovered on Mars the first extraterrestrial landscapes of likely fluvial origin, including probable catastrophic flood channels (outflow channels) of enormous proportions^1–4^. This discovery and interest in the potential for life prompted the selection of an outflow channel in the Chryse Planitia region, Maja Valles, as NASA's first landing site^5^. In 1976, the Viking 1 Lander (V1L, a.k.a. Mutch Memorial Station, https://www.jpl.nasa.gov/missions/viking-1) settled on its downstream reaches (Fig. 1a).

Early^5,8,9^ and recent^10^ remote-sensing investigations suggest that the V1L site should be on a catastrophic flood outwash deposit, generally dated as Late Hesperian (~ 3.61 to ~ 3.38 Ga)^10^. However, the landscapes imaged by the lander do not show expected megaflood features, such as imbricated boulders or streamlined islands^11^. Instead, NASA discovered that the site occurs within a boulder-strewn plain (Fig. S1), interpreted as the top of a multimeter-thick breccia^12,13^. An early attempt to explain the region's absence of fluvial features hypothesized that the site was on thick crater ejecta^14^. However, nearby craters were insufficient to account for the high boulder abundance^12^. In addition, while the deposit was then explained as extrusive and near-surface basaltic igneous rocks that were mechanically and chemically weathered, unambiguous lava fragments were found to be rare^12^. Consequently, the landing site's orbital and in-situ observations remained poorly reconciled and lacking in consensus during the V1L's 6 years of operation on Mars, giving rise to an enduring mystery in planetary exploration.

The outflow channel-forming catastrophic floods were later considered to have generated a Late Hesperian northern plains ("Deuteronilus"^15,16^) ocean^17–19^. This hypothesis prompted a subsequent mapping investigation to postulate that the V1L site could be on an ancient marine margin^20^. For the first time, that research suggested a possible connection between a Martian landing site and the proposed flood-generated ocean^15–19^. However, because the extent of the proposed breccia deposit^13^ at the V1L site was still unknown, its precise source, and therefore its possible association to the ocean, remained difficult to establish.

Here, we reinterpret the V1L site as part of a megatsunami deposit and document new insights into the ocean's evolution and history. Recent investigations suggest that megatsunamis formed within this water body^6,21–24^. Mapping by Rodriguez et al.^6^ indicates that the Chryse Planitia Highland-Lowland Boundary (HLB) plains include two deposits potentially emplaced by impact-triggered megatsunamis, which were sourced from then-undetermined locations within the northern ocean (unit members lHl_1_ and lHl_2_ in Rodriguez, et al.^6^). These potential megatsunami deposits have typical widths and lengths reaching several hundred kilometers and exhibit relief gains of a few hundred meters^6^. Hereon, we refer to these geologic deposits as the older and younger megatsunami deposits.

Rodriguez, et al.^6^ suggested that the older megatsunami propagated up from a paleoshoreline at ~ − 3800 m, and a much later one, extending from a regressed marine margin at ~ − 4100 m, emplaced the younger deposit (Fig. S2). Both paleoshoreline stands fall within the elevation range of Contact 2 (Deuteronilus level) at − 3760 m ± 560 m proposed by Parker, et al.^15^ based on the early mapping of potential paleoshoreline features and topography then available from Viking Orbiter data.

The published mapping^6^ shows that the older deposit's margin between the terminal areas of Kasei and Maja Valles is within ~ 25 km of the V1L (Fig. 1a), raising the prospect that the lander could be on the megatsunami deposit. However, exploring this hypothesis requires simulations determining the megatsunami's run-up extents from its source region plus a more detailed mapping reassessment of the landing site region.

Numerical and geologic investigations^21,22^ identified Lomonosov crater (centered at 65° 1′ N; 9° 24′ W) as a candidate for a Late Hesperian megatsunami source impact. This identification was grounded mainly on simulation work that successfully reproduced run-up distances inferred from the mapping of widespread highland-facing lobes in northeastern Arabia Terra. These lobate deposits were mapped by Rodriguez, et al.^6^ to be part of the younger megatsunami deposit.

Until now, the identification of the older megatsunami source impact has remained elusive. Recognizing marine impact craters within the Martian northern plains is challenging. Most of these crater candidates occur within Arabia Terra^25,26^; hence, their formation likely occurred due to impacts into the shallow margins of a proposed Noachian ("Arabian"^15,16^) ocean (~ > 3.7 Ga)^25,26^, significantly predating the Late Hesperian ocean. Furthermore, many northern plains craters are superimposed on the Vastitas Borealis Formation (VBF)^10,27^, an Early Amazonian (~ 3.37 to ~ 1.24 Ga) geological unit interpreted as the Late Hesperian ocean's frozen residue^28^. Consequently, most of these craters likely postdate the liquid state of the ocean. Particularly relevant to our study are long recognized, buried northern plains craters^29,30^, which, in addition to abundant Noachian populations (~ 4 Ga to ~ 3.72 Ga), could also contain a subset of Late Hesperian marine craters.

## Analyses and results

**Figure 2:**
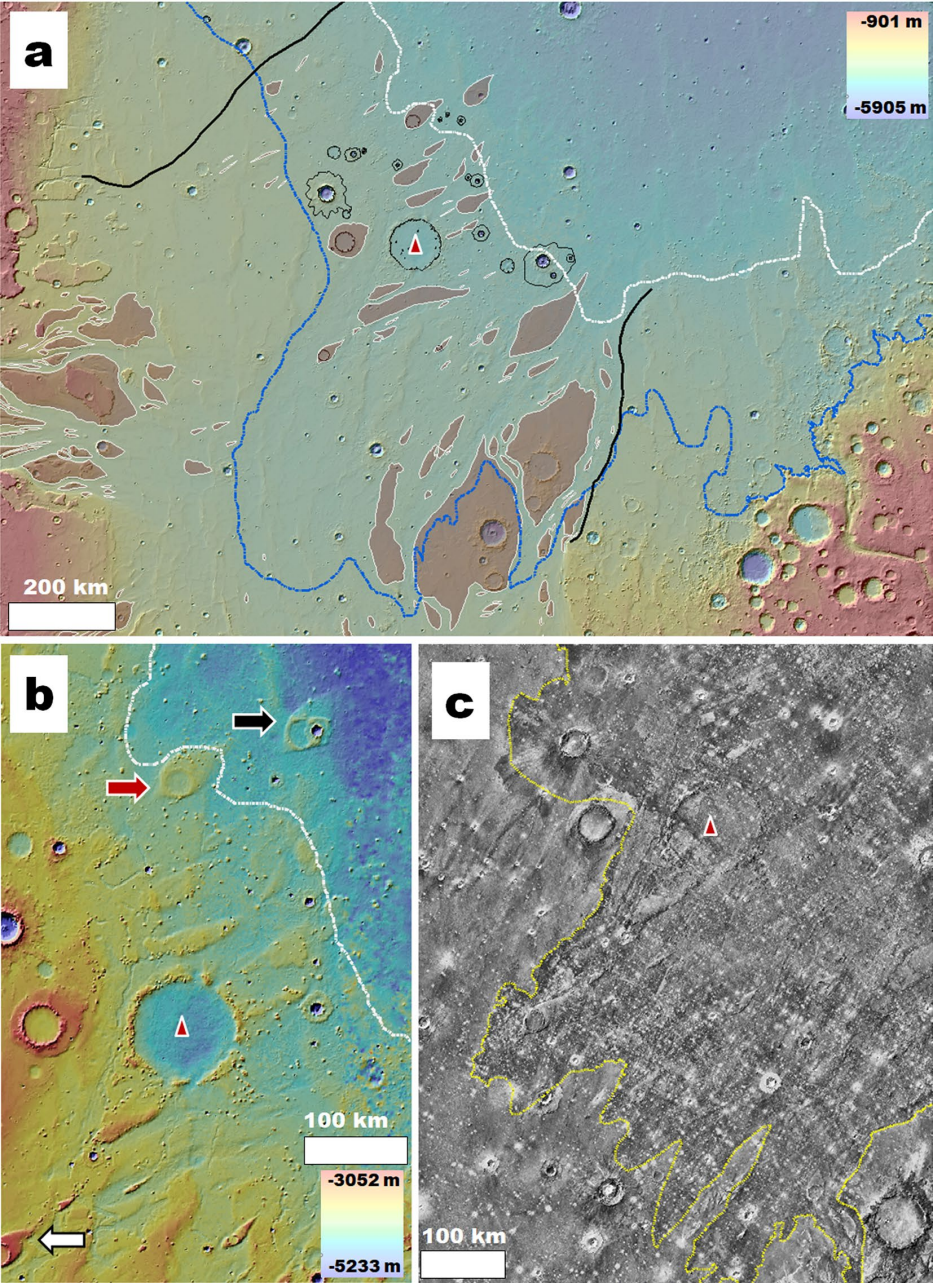
Physiographic and geologic setting of Pohl. In each panel, the crater's center is indicated by a red triangle. **(a)** View of Chryse Planitia showing widespread streamlined features (shaded brown) produced by catastrophic flooding into the northern plains. The black lines delineate the approximate lateral extents of these fluvial terrains. The white and blue dashed lines delineate the lower (− 4100 m) and upper (− 3800 m) paleoshoreline stands mapped by Rodriguez, et al.^6^. The lower of these, ~ 130 km northeast from Pohl, marks the abrupt termination of the region's catastrophic flood surface features. Combined color and shaded-relief MOLA DEMs (460 m per pixel, credit: MOLA Science Team, MSS, JPL, NASA). **(b)** Close-up view of Pohl showing that it forms a significant discontinuity of the regionally scoured landscapes' topography. The black and white arrows identify streamlined features downstream and upstream from Pohl. The white dashed line traces the lower shoreline (~ − 4100 m) mapped by Rodriguez, et al.^6^. The red arrow identifies the streamlined island shown in Fig. S3c. Combined color and shaded-relief MOLA DEMs (460 m per pixel, credit: MOLA Science Team, MSS, JPL, NASA). **(c)** View showing the younger megatsunami deposit regional upper reaches covering and embaying Pohl (unit member lHl_2_ in Rodriguez, et al.^6^). The deposit's surface appears dark in this nighttime thermal infrared image, and a yellow dotted line delineates its upper boundary. THEMIS nighttime IR global layer (http://www.mars.asu.edu/data/, 100 m per pixel, credit: Christensen, et al.^7^). We produced this figure using Esri's ArcGIS 10.3 (http://www.esri.com/software/arcgis).

**Figure 3:**
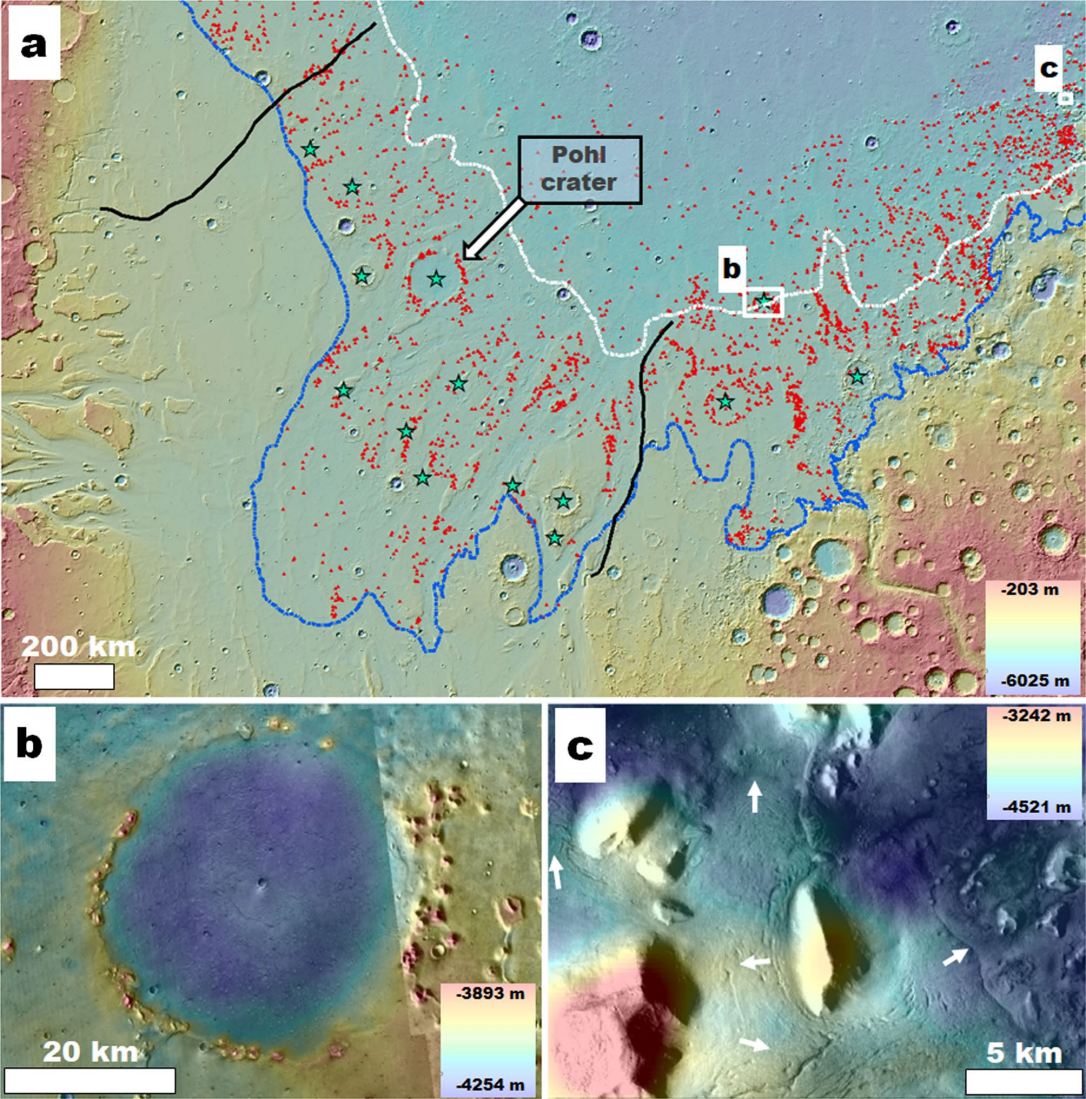
**(a)** View of Chryse Planitia showing the upper and lower paleoshoreline stands (blue and white lines, respectively). The black lines mark the margins of the regional surfaces affected by outflow channel dissection. The red symbols identify numerous knobs forming an extensive field within and north of a proposed paleo-oceanic area of regression from the higher to the lower paleoshoreline. Pohl is one of the field's several knobby-rim craters (green star symbols). Color MOLA DEM (460 m per pixel, credit: MOLA Science Team, MSS, JPL, NASA) over a THEMIS nighttime IR global layer (http://www.mars.asu.edu/data/, 100 m per pixel, credit: Christensen, et al.^7^). **(b)** Close-up view of one of the knobby-rim craters located outside the areas of outflow channel dissection. The knobs, such as observed at Pohl, exhibit no flow-related streamlining, suggesting that catastrophic flood erosion did not lead to the field's formation. Color-coded shaded-relief MOLA digital elevation model (460 m per pixel, credit: MOLA Science Team, MSS, JPL, NASA) over part of a CTX mosaic (6 m per pixel, credit: NASA/JPL/Malin Space Science Systems (https://www.msss.com/mro/marci/images/tips/mediatips.html) and a THEMIS daytime IR global layer (http://www.mars.asu.edu/data/, 100 m per pixel, credit: Christensen, et al.^7^). **(c)** View of possible debris-covered glaciers with evidence of multiple directional flow patterns (e.g., white arrows) around and along the margins of knobs and mesas within the northeastern-most part of the mapped field, suggesting that glacial erosion contributed to its formation. Color-coded shaded-relief MOLA digital elevation model (460 m per pixel, credit: MOLA Science Team, MSS, JPL, NASA) over part of a CTX mosaic (6 m per pixel, credit: NASA/JPL/Malin Space Science Systems (https://www.msss.com/mro/marci/images/tips/mediatips.html). We produced this figure using Esri's ArcGIS 10.3 (http://www.esri.com/software/arcgis).

**Figure 4:**
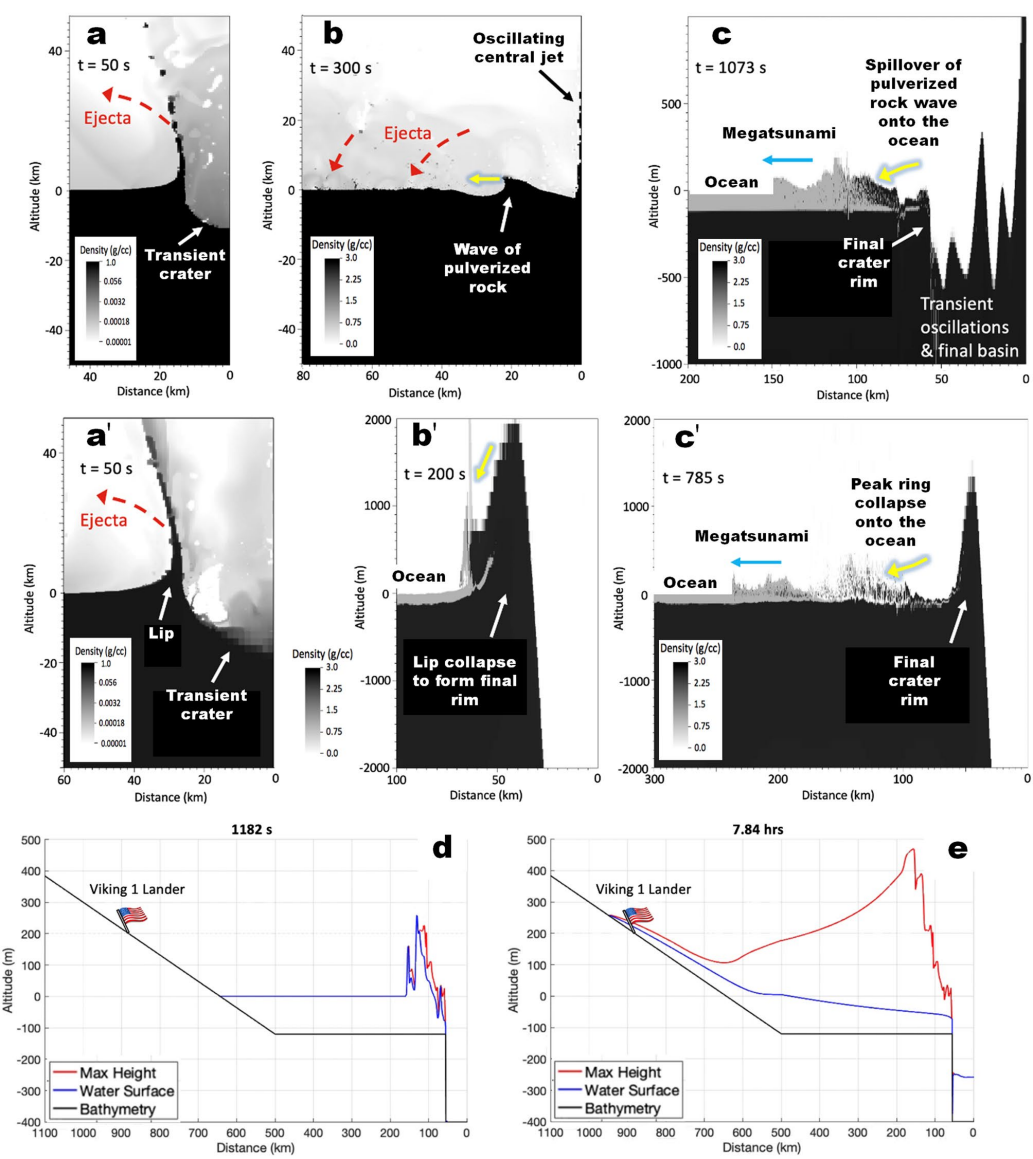
Megatsunami simulations of impacts into the northern ocean (120 m deep). **(a–c)** Simulation frames showing an impact into the weaker ground (supplementary movies 2 & 3). An asteroid 3 km in diameter (impact velocity: 10.6 km/s; density: 2.63 g/cc) is required to form Pohl. Details on the strength models are given in the supplementary material. The lack of residual strength results in the formation of a large Worthington^31^ central jet of pulverized rock and the generation of transient oscillations, pushing the crater rim to about twice the diameter of the initial transient crater. The pulverized rock "waves" spilling over the incipient rim **(b,c)** trigger a megatsunami **(c)**, and the collapsing ejecta curtain **(b)** generates debris-laden water. **(a'–c')** Simulation frames showing an impact into the stronger ground, in which a 0.01 MPa residual strength of pulverized rock inhibits the development of a central jet of pulverized rock and transient oscillations (supplementary movies 5 & 6). The final crater extends slightly beyond the initial transient crater as the base of the ejecta curtain (lip) collapses to form the peak ring. Consequently, in this case, a 9 km asteroid (impact velocity: 10.6 km/s; density: 2.63 g/cc) is required to form Pohl. In this scenario, the collapse of the incipient peak ring on the surrounding ocean drove the megatsunami. In addition to the ejecta fallout, we consider that both the collapse of the central jet and rim materials could have contributed to the megatsunami's densification. **(d,e)** 1-D GEOCLAW simulation using the shallow water solver for long-range propagation, onshore run-up, and an idealized bathymetry from Pohl crater to the V1L site. Panel **(d)** shows the megatsunami transfer of the megatsunami from the impact simulation into weaker ground to the shallow water solver. Panel **(e)** to the shallow water solver, which shows that the megatsunami initially reached ~ 500 m in height and propagated to ~ 250 m above sea level, overrunning the V1L site. Figure 5 shows 2-D simulations for both megatsunamis accounting for varying topography. Profile and horizontal velocity data is available in the supplementary material.

**Figure 5:**
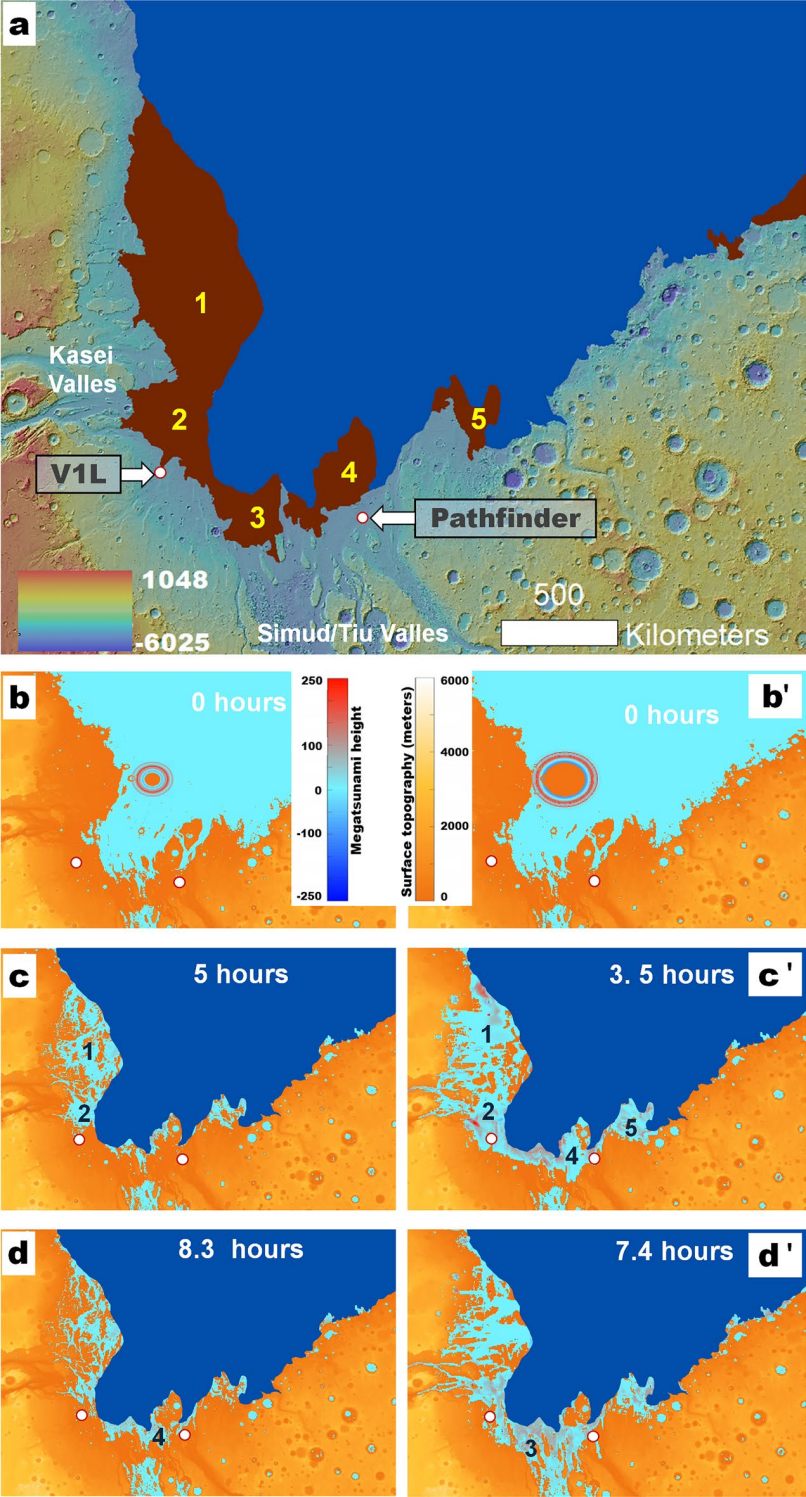
**(a)** View of the Chryse Planitia region. The dark blue area is a reconstruction of the northern ocean with a paleoshoreline at − 3800 m. The adjoining brown areas show the mapped older megatsunami areas of overland deposition (lHl_1_) mapped by Rodriguez, et al.^6^. The locations of the V1L and the Mars Pathfinder (MPF) Lander are indicated. Numbers 1–5 identify the megatsunami's largest lobes. **(b–d**) Weak  Ground (WG) simulation (zero residual shear strength), 3 km asteroid (supplementary movie 1). **(b'–d'**) Strong Ground (SG) simulation, (0.01 MPa residual shear strength), 9 km asteroid (supplementary movie 4). The simulations show megatsunami propagation directions and extents that closely match the mapping shown in panel (**a**). Both simulations predict inundations over the V1L site as well as close to (WG simulation) or over (SG simulation) the Pathfinder site. All the panels use MOLA DEM bases (460 m per pixel, credit: MOLA Science Team, MSS, JPL, NASA). The topographic ranges in the DEMs shown in the simulation panels (**b–d**, **b'–d'**) represent the regional relief with a zero-meter base. We produced this figure using Esri's ArcGIS 10.3 (http://www.esri.com/software/arcgis).

**Figure 6:**
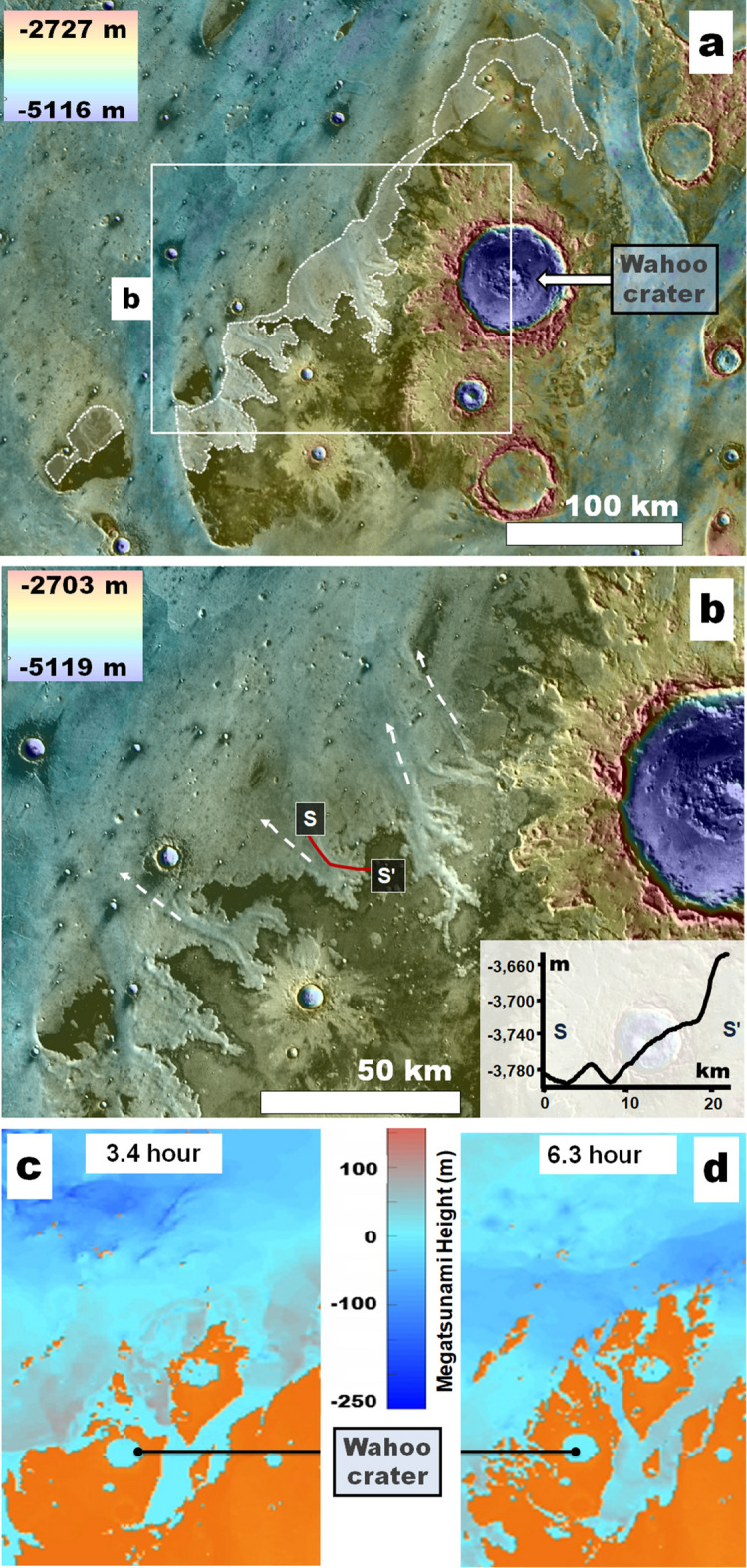
**(a)** View of an extensive streamlined mesa forming the base of Wahoo crater in Chryse Planitia (Wahoo crater is identified in Fig.1a). The dashed white lines demark channeled slopes facing towards the northern plains. These are mostly not connected upstream to outflow channels. **(b)** Close-up view on panel (**a**) showing some of the channels (dashed white lines). The elevation profile S-S' along one of the channels shows a ~ 140 m relief gain ~ over 20 km (~ 0.4°). Panels **(a,b)** Color MOLA DEM (460 m per pixel, credit: MOLA Science Team, MSS, JPL, NASA) over a THEMIS daytime IR global layer (http://www.mars.asu.edu/data/, 100 m per pixel, credit: Christensen, et al.^7^). **(c,d)** Parts of simulation in Fig. 5 showing WG movie 1, timesteps 3.4 and 6.4 h. During the time interval, the megatsunami recedes from the Wahoo crater's northern margin. The predicted backwash happens where the noted channels occur. We produced this figure using Esri's ArcGIS 10.3 (http://www.esri.com/software/arcgis).

**Figure 7:**
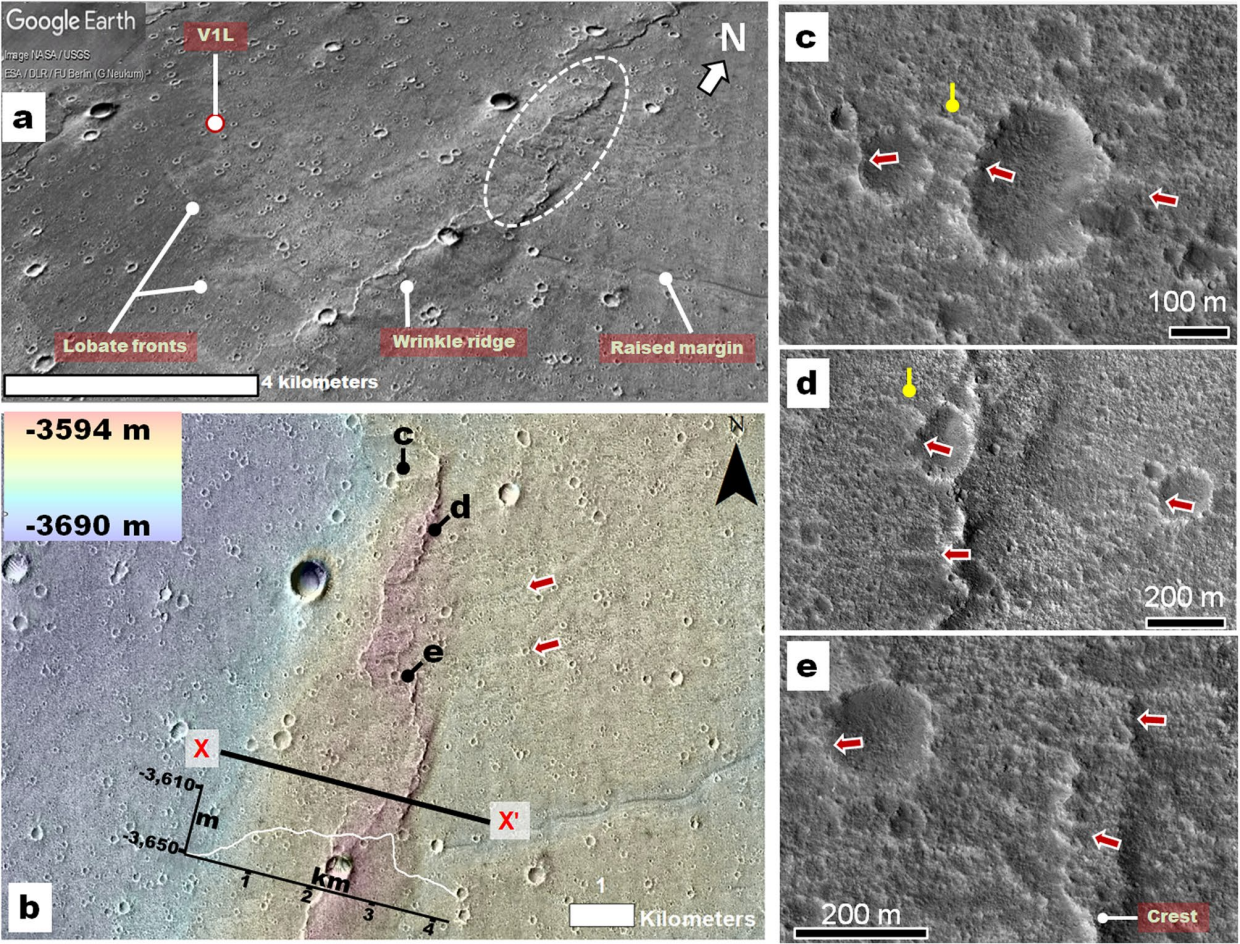
**(a)** CTX perspective view showing the V1L site (red-circled white dot) along the uppermost fringes of the remapped older megatsunami marginal deposit (V1LD, in Fig. 1b; Fig. 1a shows the deposit's original mapping^6^). Features diagnostic of run-up flow include (1) a scoured wrinkle ridge segment (dashed ellipse) separating (2) highland-facing lobate smooth areas and (3) a lower, scoured deposit section with raised margins. The image is rotated for better viewing (see north arrow orientation). This terrain assemblage is consistent with a megatsunami lobe propagating and decelerating over a wrinkle ridge, generating localized erosion on the ridge and lobate deposits beyond it. **(b,** north is up**)** Close-up of the region within the dashed ellipse in panel **(a)**. The region includes west-trending lineations (red arrows) consistent with scouring by a unidirectional flow that started within the northern plains. The topographic profile X-X' shows a relief measurement of the wrinkle ridge crest (~ 40 m), providing an approximation of the local overflowed topography. The image is a Digital Terrain Model (DTM) created from CTX images G02_018887_2026 and J04_046170_2025 (~ 24 m post spacing) using the Ames Stereo Pipeline superimposed on associated CTX orthorectified images (6 m per pixel, credit: NASA/JPL/Malin Space Science Systems (https://www.msss.com/mro/marci/images/tips/mediatips.html). **(c–e, north is up)** HiRISE ESP_072321_2025 (25 cm per pixel), close-up views at and near the proposed scoured section of the wrinkle ridge crest. The red arrows identify possible dissection marks, some which cut into the crest, as shown in panel **(e)**. The presence of craters with dissected rims (e.g., yellow pointers) indicates that the flow must have been extremely energetic and mostly erosional over the basement uplifted during wrinkle ridge formation. We produced this figure using Esri's ArcGIS 10.3 (http://www.esri.com/software/arcgis).

In this section, we present (1) geological evidence of an impact crater holding the stratigraphic and spatial characteristics expected for an older megatsunami's source impact (Figs. 2, 3), (2) numerical models of this crater's impact-generated megatsunami wave (Figs. 4, 5), and (3) geologic observations consistent with the modeled numerical predictions (Figs. 1b, 4a, 6, 7).

### Morphologic mapping approach

To produce our broad geologic characterizations and maps, we utilized the publicly available global seamless Mars Reconnaissance Orbiter (MRO) Context Camera (CTX)^32^ (5.6 m per pixel) visible light mosaic from the Murray Lab^33^ (http://murray-lab.caltech.edu/CTX/), and the Mars Odyssey Thermal Emission Imaging System (THEMIS)^34^ nighttime and daytime infrared image mosaics^35^ (100 m per pixel), in combination with Mars Global Surveyor (MGS) Mars Orbiter Laser Altimeter (MOLA)-Mars Express (MEX) High-Resolution Stereo Camera (HRSC) blended Digital Elevation Model (DEM, 200 m per pixel)^36^. We also produced multimeter-scale geologic characterizations using images from the Mars Reconnaissance Orbiter's High-Resolution Imaging Science Experiment^37^.

We performed our mapping at a 1:750,000 scale using Esri's ArcGIS 10.3 (http://www.esri.com/software/arcgis). We mapped the sedimentary unit where the V1L site is located (Fig. 1b). The CTX visible light mosaic from the Murray Lab did not exist when Rodriguez, et al.^6^ conducted their regional survey using lower resolution THEMIS IR. To determine key stratigraphic relationships utilized in our geologic reconstructions, we mapped (1) the streamlined features of Chryse Planitia (Fig. 2a) and (2) significant knob clusters mainly situated between the upper and lower paleoshoreline stands (i.e., − 3800 and − 4100 m^6^) (Fig. 3a). The number and spatial distribution of knobs between the two paleoshorelines are generally much greater than above the higher one. However, relatively less extensive clusters of knobs are scattered over some highland sections above some upper paleoshoreline sections. These are Noachian features connected to a much older resurfacing^38^ and are excluded from our mapping. To avoid cluttering the figure, we only mapped enough knobs between the shoreline elevations to identify their clustering and widespread distribution.

### A Late Hesperian marine crater in the Chryse Planitia region

We mapped the distribution of streamlined mesas within Chryse Planitia, a region of previously recognized catastrophic flood convergence into the northern plains^10,27^. Our mapping shows that these areas comprise Mars' most extensive landforms due to catastrophic flooding, with a maximum width of ~ 1300 km from the northwestern margin of Kasei Valles to the southeastern margin of Ares Valles (Fig. 2a). Furthermore, these terrains form a seaward extension of the circum-Chryse outflow channels (Fig. 2a), hence, highlighting the former existence of a potential submarine platform submerged below the proposed ~ -3800 m Late Hesperian sea level (Parker, et al.^15^, Rodriguez, et al.^6^, Fig. S2).

Within this platform, we identified a ~ 110-km-diameter impact crater centered at 34° 3′ N; 37° 1′ W (Figs. 1a, 2a,b), recently named Pohl by the International Astronomical Union (IAU) Working Group for Planetary System Nomenclature (WGPSN). The impact locally obliterated the outflow channels and, consequently, postdates them and the northern plains' oceanic inundation produced by their cataclysmic floods. In addition, the younger megatsunami deposit (Fig. 2c) embays and superposes the crater's rim and covers most of the proposed marine platform. These stratigraphic constraints allow us to place the impact event between the cessation of ocean-forming floods and the ocean's regression to the − 4100 m paleoshoreline, implying that it most likely occurred into the ocean.

Our inferred stratigraphic relationships also suggest that Pohl could have experienced resurfacing connected to the proposed − 3800 m to − 4100 m marine regression (Fig. S2)^6^. The crater's knobby ridge (Figs. 2b; S3a,b) is part of a vast knobby field (hereon, Pohl knobby field) between these paleoshoreline stands (Fig. 3a). The field contains other similarly degraded crater rims beyond Chryse Planitia's catastrophic flood-dissected areas (Fig. 3a,b), suggesting regionally extensive post-catastrophic flood resurfacing. In addition, knob clusters locally superpose younger megatsunami-covered streamlined mesas, indicating that knob development persisted after the younger megatsunami's emplacement (e.g., red arrow in Fig. 2b; white arrow in Fig. S3c). Hence, we propose that the modification of Pohl's rim into a knobby topography was part of protracted regional history that involved widespread, episodic coastal erosion and deposition. Furthermore, numerous pitted cones (i.e., possible mud volcanoes^39^)﻿ superpose younger megatsunami surfaces at and near Pohl (e.g., Fig. S3e). Consequently, sedimentary extrusions could have also contributed to the knobs' generation. 

### Simulating Pohl's impact-triggered megatsunami

Our northern plains survey recognizes Pohl as the only candidate crater with a regional stratigraphy suggestive of an impact history that could have produced the older megatsunami. Here, we present simulation studies that further test this hypothesis.

#### Numerical tools and simulation grid setup

The impact cratering simulations and initial megatsunami formation used the ALE3D hydrocode^40^ from Lawrence Livermore National Laboratory (accessible to the U.S. Government only). Similar results should be obtainable with another hydrocode (e.g., iSALE^41^, accessible to academia). Our simulations used a fixed mesh (Eulerian) and are 2-D axisymmetric, which, while significantly less computationally taxing than a 3-D approach, restricts the impact to a vertical entry. The mesh has a 10 m resolution on the water surface and increases with distance from sea level so that the resolution is approximately 1/10th of the altitude or depth out to a maximum cell size of 500 m. The radius of the impact simulation domain is 300 km.

#### Choice of paleophysiographic and atmospheric parameters

Our simulation setup adopted a paleoshoreline at − 3800 m, positioning the formation of Pohl into a shallow (~ 120 m deep) part of the Late Hesperian northern ocean. Previous publications support the validity of this choice of sea level. For example, Rodriguez, et al.^6^ found that the − 3800 m elevation forms the lowest extents of the oldest megatsunami lobes and superposed backwash channels. This elevation also matches the average elevation of Contact 2 (i.e., − 3760 m ± 560 m) proposed by Parker, et al.^15^. Numerical models^42^, while still debatable^43^, suggest the formation of Tharsis and true polar wander did not significantly warp the HLB region where the V1L is located, further validating the paleoshoreline's likely accuracy.

Other potential Hesperian paleoshorelines at higher elevations, for example, as suggested by Duran, et al.^44^, might indicate a more extensive oceanic phase within the northern plains. However, there are no paleoshorelines superposed over the older megatsunami deposit, suggesting that those possible higher paleoshoreline stands likely predated its emplacement.

The atmosphere was modeled as carbon dioxide in hydrostatic equilibrium up to an altitude of 72 km, with a surface pressure of 0.1 bar and an isothermal temperature of 283K^45^. The 0.1 bar surface pressure falls within a previously predicted range of 0.04–1 bar^45,46^. However, a different value would have likely caused negligible variation in the crater size because even 1 bar is much less than the 4.5 bar at 120 m depth of water on Mars and 2400 bar at 20 km depth of basalt at maximum transient crater depth. The density of even 1 bar CO_2_ is about 0.1% that of liquid water, so the air pressure would also have a negligible effect on the megatsunami propagation, which is just a gravity driven interfacial wave.

#### Determining sensitivity of megatsunami characteristics to impact parameters

The choice of impact parameters (e.g., ground properties, impactor size, speed, density) is essential in determining the dimensions of a crater and megatsunami characteristics. To investigate the sensitivity of megatsunami sizes and run-ups to impact parameters, we ran two simulations into a weak ground model and two into strong ground. The simulations extended 100 km deep, so the mantle, below 120 km deep, was not modeled. The assumed geotherm was linearly interpolated between 283 K at the surface and 1650 K at 120 km.

In the weak and strong ground models, the residual shear strength of the crushed rock is zero and 0.01 MPa, respectively (see supplementary materials for further details). The two weak ground simulations assumed a 3-km-diameter asteroid (2.63 g/cc, an average value for ordinary chondrite, https://neoproperties.arc.nasa.gov/) with a vertical impact velocity of 10.6 km/s, an average asteroid impact speed on Mars^47^:

*Case 1* 60-km-thick crust (2.8 g/cc) over 3.2 g/cc upper mantle^48^.

*Case 2* 120-km-thick basalt lithosphere (3.2 g/cc)^49^.

The two strong ground simulations assume that the entire 120-km-thick basalt lithosphere had a 3.2 g/cc density^49^.

*Case 3* 9-km-diameter asteroid (2.63 g/cc) with a vertical impact velocity of 10.6 km/s^47^.

*Case 4* 10.132-km-diameter icy comet (0.5 g/cc; = 50% porosity ice, without any dense minerals) with a vertical impact velocity of 21 km/s^50^.

Each simulation was briefly run implicitly to establish equilibrium, after which the impactors were added, and the simulations were then run with explicit timesteps. After some violent oscillations, the impact-generated megatsunami waves settled down into shallow water waves, traveling with wavelengths much greater than the water depth and a horizontal particle velocity that is uniform with depth to the seafloor (supplementary files of WG (Weak Ground) and SG (Strong Ground) water surface and horizontal particle velocity). The uniformity with depth means the shallow water equations can be solved as a surface wave, orders of magnitude faster than continuing the hydrocode^51^.

At this point, the water surface and horizontal particle velocity were extracted from the hydrocode simulations and used to input the shallow water solver (Fig. 4, supplementary movies and supplementary files of WG and SG water surface and horizontal particle velocity). Finally, the shallow water equations were solved using the GEOCLAW package^51^, downloadable from https://www.clawpack.org/.

The strong ground models do not form the flat-bottomed basins seen in craters with dimensions similar to Pohl's; therefore, additional physics, such as acoustic fluidization, is often invoked to temporarily reduce the residual strength of failed material so it can flow down from the crater walls. In this simulation, we neglected acoustic fluidization since it is the megatsunami outside the crater that is of interest. Furthermore, the seafloor friction can be a tuning parameter to adjust the run-up distance for a given tsunami. Seafloor friction depends, for example, on the cohesiveness of the seafloor, velocity and turbulence of the tsunami, debris entrained in the flow, and gravity. In these simulations, it was set to a minimal value due to the lower gravity on Mars, but it also remains an item for future investigation.

#### Selecting the simulations

We find that both weak ground impacts produced similar megatsunamis and that those generated in the two strong ground impacts were almost identical. These simulations show that ground strength, especially the residual shear strength of pulverized rock, strongly affects the size and energy of the impactor required to produce the craters. However, the similarity of the resulting megatsunamis demonstrates that the crater diameter is a key control on waves' characteristics, including run-up extents (supplementary movies).

For the same ground model, various impactor size, density, impact velocity, and angle combinations could have delivered similar energy and momentum, producing the same size crater. In such a case, differences in megatsunami sizes would have likely resulted from varying magnitudes in the collapse and the overflow of crater rim material.

In this article, we present and discuss results concerning cases 1 and 3 above. Hereon, we refer to these cases as Weak Ground (WG) and Strong Ground (SG) simulations. Both formed craters 110 km in diameter, hence, matching the size of Pohl. The crater's formation in the strong ground required a 9 km asteroid carrying ~ 5.6 × 10^22^ J = 13 million megatons of TNT energy. On the other hand, its formation in the weak ground resulted from a 3 km asteroid releasing ~ 2.1 × 10^21^ J = 0.5 million megatons of energy.

### Pohl as the source of the older megatsunami

Our simulations reveal megatsunami run-ups with excellent general correspondence to the mapped megatsunami margins (Fig. 5). Their spatial agreement occurs over ~ 4000 km of topographically diverse HLB terrains, including reconstructed coastal areas as close as ~ 200 km and as far as ~ 1500 km from the center of the impact site, all of which support the formation of Pohl as the older megatsunami's source. The WG model returned an almost complete match (Fig. 5, supplementary WG movies 1–3). While the SG simulation also produces run-up distances that approximate the mapping results, they also predict significant landward inundations within Kasei Valles and Simud/Tiu Valles (Fig. 5; supplementary SG movies 4–6).

### Viking 1 landing site reinterpretation and Pathfinder landing site considerations

Our 1D and 2D simulations indicate that the distal extent of the megatsunami reached the V1L site, nearly 900 km from the impact (Figs. 4, 5; supplementary WG movies 1–3, SG movies 4–6), suggesting the potential presence of run-up materials. We find that the V1L site occurs within a previously unrecognized broad deposit, which we refer to as the Viking 1 Lander Deposit (V1LD, Fig. 1b, Fig. S4). The deposit's highland-facing margin indicates that it might be a run-up lobe. A channel extending from Kasei Valles separates the V1LD's base from the older megatsunami deposit as initially mapped in 2016^6^. Hence, we consider that, before this channel's formation, the V1LD comprised the older deposit's upper boundary (Fig. 1b, Fig. S4) and attribute its origin to later dissection. Catastrophic floods postdating the older megatsunami would have preferentially flowed through the lowest, most deeply dissected floors of Kasei Valles (Fig. S4). However, the older megatsunami deposits intruding these floors lack indications of post-emplacement overflow or dissection (Fig. S4).

Alternatively, the dissection resulted from megatsunami backwash. Such a backwash pattern is predicted in our WG simulation to have extended from the southern terminal area of Kasei Valles (Fig. S5; supplementary WG movie 1: timestep 4 to 5 h). A similar backwash trend is also present in the SG simulation (supplementary SG movie 4: timestep 4.2 to 6.3 h). Although evidence of backwash in Chryse Planitia does not appear to be widespread^6^, we also recognize the presence of abruptly-appearing channels at an area of simulated backwash along the northern plains-facing margins of a broad mesa where Wahoo crater is located (Fig. 6; supplementary WG movie 1: timestep 2.5 to 4.5 h), thereby reinforcing the simulations' accuracy. In the context of these observations and simulations, we suggest that the V1L site's bouldery plains comprise the older megatsunami's upper reaches.

The SG simulation also predicts megatsunami inundation zones, including at the Pathfinder site (Fig. 5; supplementary SG movie 4: after timestep 3.3 h). The regional megatsunami inundation raises the possibility that its backwash flows could have contributed to the emplacement of imbricated boulders near this landing site, previously exclusively attributed to catastrophic floods^52^. In addition, the simulated megatsunami also reaches multiple large basins in southern circum-Chryse, suggesting that its ocean-displaced waters could have sourced interior regional lakes or an inland sea^53^.

#### Regional evidence of megatsunami run-up at the Viking 1 landing site

The V1L sits on the margin of a broad, lobate front (Fig. 7a), forming the uppermost reaches of the V1LD (Fig. 1b, Fig. S4). The lobe extends westward from a scoured section of a wrinkle ridge that regionally intersects the V1LD orthogonally to its overall westward orientation (Fig. 7a). The scour marks include lineations extending downhill over an elongate section of the VL1D (Fig. 7a,b). However, these do not appear to significantly mark the lobe's surface. These observations are consistent with an uphill, fast-moving flow, which decelerated as it overran and eroded the wrinkle ridge.While the energy dissipation during the wrinkle ridge overflow would have resulted in dominantly erosional features, some lineations may include deposits from when the flow's energy and carrying capacity diminished.

At multimeter scales, the wrinkle ridge's scoured zone includes aligned ridges and grooves (Fig. 7c–e). Some grooves transect craters, demonstrating that unidirectional erosion contributed to their formation. Furthermore, local curvilinear erosional patterns (e.g., yellow pointers in Fig. 7c,d) suggest topographic modulation of flow directions, consistent with dissection by fluids.

We suggest that the regional landscape at the V1L site retains terrains connected to the older megatsunami's emplacement. The 2D megatsunami simulation uses the MOLA DEM base topography. Hence, the hydrodynamics that it predicts should have higher accuracy at broader spatial scales. However, a simulation indicates some southwest-trending flows at and near the V1L site (supplementary WG movie 1: timestep 5.3 to 6.4 h).

In the context of this interpretation, we propose that the megatsunami decelerated because the wrinkle ridge (~ 40 m in relief, Fig. 7b) partially obstructed its propagation, thereby leading to the lobe's emplacement. Thus, we interpret the rocky plains recognized in images returned by the V1L in the 1970s^12,13^ as megatsunami bedload materials. Furthermore, our hypothesis offers an explanation for the boulders' high and puzzling diversity identified in those early images^12^.

### The formation of megatsunami debris flow fronts

**Figure 8:**
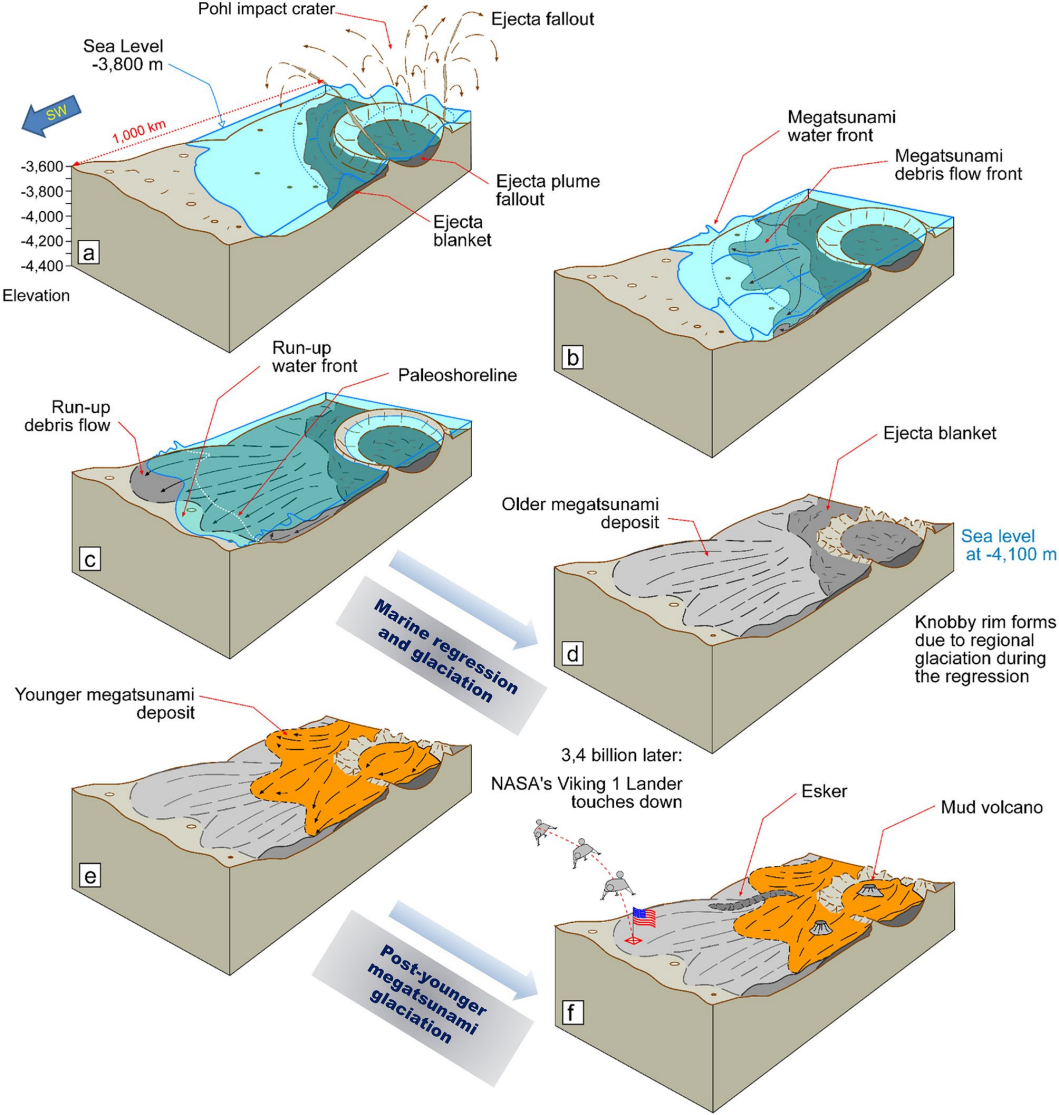
Schematic reconstruction showing the history of megatsunami formation and modification in the Chryse Planitia region. **(a)** Pohl crater forms within a shallow marine environment, **(b)** triggering megatsunami water and debris flow fronts. **(c)** The wave fronts extensively inundate the highland lowland boundary plains, including a section ~ 900 km southwest of the impact site. **(d)** The ocean regresses to ~ − 4100 m, accompanied by regional glacier dissection, which erode the rims of Pohl and other craters. **(e)** The younger megatsunami overflows Pohl and parts of the older megatsunami. Glaciation continues, and mud volcanoes later source and emerge from the younger megatsunami deposit. **(f)** ~ 3.4 billion years later, the Viking 1 Lander touches down on the edge of the older megatsunami deposit.

Our multimeter scale remote sensing observations reveal that the lobe is a poorly sorted, bouldery deposit (Fig. S6), which is consistent with the lander observations^12,13^. The boulder-rich bedload's emplacement into a lobate deposit suggests propagation as a coherent, unidirectional fluidized mass movement, which we interpret as a run-up debris flow. Here, we assess various mechanisms for the generation of debris flow fronts within the older megatsunami's wave train. Our 1D simulations show that, from its initiation, the megatsunami height exceeded the ocean depth (Fig. 4; supplementary WG movies 2, 3; SG movies 5, 6). Consequently, it would have been turbulent and erosive from the start. While turbulent dissipation was not modeled in shallow water equations, the equations we used have been shown to reasonably predict the run-up of breaking waves and tsunamis on shorelines^54^. Turbulence on the leading edge could have eroded and transported abundant sediment, probably generating catastrophic debris flow fronts within the megatsunami (Fig. 8a-c).

Significant sources of wave-densifying sedimentary sources could have included Pohl's ejecta (Fig. 4b,c) and collapsed rim materials (Fig. 4c'), both potentially contributing to the megatsunami waves. Furthermore, the impact likely generated quakes with vertical accelerations greater than Mars' gravity (3.721 m/s^2^) to distances extending up to 150 km from the impact rim. The powerful seismicity could have injected some seafloor materials into the megatsunami, thereby also increasing its bedload (supplementary SG movie 6, Fig S7).

Fluxes in bedload acquisition could have produced significant density zonation within the megatsunami, ranging from water to debris flows (some icy) of varying rheologies and variable sediment types. We propose that a debris flow front emplaced the V1LD; therefore, the megatsunami sedimentary setting around the V1L could potentially include geologic materials derived from distant ocean floor terrains present along its propagation pathway.

## Discussion

### Absence of widespread backwash channels in Chryse Planitia megatsunami deposits

The older megatsunami did not produce widespread backwash channels in the Chryse Planitia region^6^. Two critical factors likely contributed to this regional sparsity: (1) Our simulations (supplementary WG movie 1; SG movie 4) consistently indicate that the furthest megatsunami inland inundation occurred within outflow channel interiors. Backwash flowing along the channels would have produced erosional features aligned to catastrophic flood features (i.e., towards the northern plains), thus complicating their recognition. Hence, regional backwash flow directions and distribution can be most readily assessed where these flows formed outside outflow channels.

(2) The backwash flow rate correlates to the megatsunami depth at their uppermost reaches and the average slope between these reaches and the paleoshoreline. Our simulations, which track the megatsunami depth at each timestep, indicate that the uppermost reaches are generally shallower than 10 m (Fig. S8). In addition, the average slopes are shallow (e.g., ~ 0.1 degrees between the V1L site and its nearest − 3800 m paleoshoreline). Consequently, the maximum backwash flow rates would have been a few meters per second, which would be insufficient to remobilize the boulder-rich megatsunami deposits and form networks of deeply excavated backwash channels in these materials. In other words, the run-up and backwash are asymmetric in their corresponding flow speeds and energies available to transport sediment.

### History of megatsunami deposit modifications in the Pohl knobby field

The morphogenetic disconnect between the Pohl knobby field and catastrophic flood erosion calls for an alternative formational mechanism affecting the area intervening the two paleoshoreline stands (i.e., from − 3800 to − 4100 m). In this section, we do not propose a single origin to all the knobs within the field; instead, we consider mesas and hills, formed during circum-oceanic glaciation lasting and postdating the paleoshoreline regression, and later megatsunami deposit-sourced mud volcanoes as possible contributors.

#### Late Hesperian circum-oceanic glaciation in Chryse Planitia

We find that along the Pohl knobby field northeastern margin (Fig. 3), there are lobate aprons, surrounding knobs, and mesa clusters. These aprons are generally considered to be debris-covered glaciers^55–61^, and the presence of multi-directional flow fronts (e.g., white arrows in Fig. 3c) is consistent with the intersecting dissection patterns necessary for the generation of the knobby topography.

In addition, the younger megatsunami deposit in the vicinity of Pohl exhibits superposed low, linear to gently sinuous troughs containing ridges (e.g., Fig. S3d). These features are possible subglacial "tunnel channels" and medial eskers. Similar feature interpretations throughout the northern plains^62,63^ are consistent with resurfacing due to wet-based glacier erosion and deposition^63–66^. On Earth, eskers form when highly pressurized water erodes subglacial channels both into the bed and upward into the ice. As hydraulic pressure declines, the ice tunnel starts to close plastically. Finally, glacio-fluvial sediment (an esker) is deposited in the remaining narrower tunnel.

Therefore, we hypothesize that Late Hesperian circum-oceanic glaciation^6,63,67,68^ may have contributed to the knobby field. The glaciated belt probably developed during the − 3800 m to − 4100 m marine regression^6^ and persisted after the younger megatsunami deposit's emplacement (Fig. 8d–f).

Kreslavsky and Head^28^ and Kargel et al.^63^ argue that the northern ocean could have frozen into a stable, long-lasting residue. In agreement with their hypothesis, our mapping reveals the abrupt termination of the Chryse Planitia's fluvial landscapes at the lower paleoshoreline elevation (Fig. 2a) (~ − 4100 m^6^), suggesting that the ocean's region-wide stabilization and end of its regression probably happened at this elevation. Likewise, circum-oceanic ice sheets could have stabilized long-term so that some of their residues still exist (or existed until recently). In the context of their possible protracted stabilization, we interpret that the glaciers' surface ages ranging from the Late Hesperian (~ 3.61 to ~ 3.38 Ga^10^)^69^ to the Late Amazonian (~ 0.328 Ga to the present time)^70,71^ might, at least in some cases, represent stages of episodic flow and elevated sublimation and not necessarily their time of emplacement.

#### Megatsunami deposits as a possible source of mud volcanism

The Pohl knobby field includes widespread pitted cones previously interpreted as possible mud volcanoes^39^. These cones form large clusters on (1) the younger megatsunami deposit (Fig. S9), including an extensive occurrence within the floor of Pohl (e.g., Figs. S3b,e; S9), and (2) the hypothesized lower-lying frozen ocean (see the termination of streamlined mesas in Fig. 2a) (Fig. S9). In contrast, the clusters are absent from sampled older megatsunami areas (Fig. S9). Hence, we propose that the long-term retention of voluminous, briny seawater frozen during the younger megatsunami's run-up, as postulated by Rodriguez, et al.^6^, could have facilitated their formation and high regional abundance (Fig. 8f). Furthermore, the bimodal clustering distribution (i.e., on the younger megatsunami deposit and frozen ocean residue) is also consistent with long-term mud volcanism, suggesting a protracted eruptive history that probably postdated the oce﻿an's freezing. It is also possible that ﻿the deposit's rapid, post-emplacement compaction contributed to regional mud volcanism^24^. However, we identified possible mud volcanoes partly covering regional polygonal trough sections (e.g., Fig. S3e), indicating a formation that must have long postdated the megatsunami's emplacement and its early tectonic modifications (Fig. 8f). Hence, we propose that a sizable subset in their population was likely connected to a gradual, multi-stage formation history.

### Paleoenvironmental and astrobiological considerations

#### Marine deposits at the Viking 1 Lander site as constraints to the ocean's chemistry

The V1L chemistry and imaging experiments suggested that soil clods are largely phyllosilicates, including nontronite, lightly cemented by sulfate, chloride, and carbonate salts^72^. The clods are presumably young sedimentary aggregations; however, their source and compositional makeup could be ancient. A possibility is that they are composed of a combination of global aeolian dust^73^ and volcanic exhalative alteration phases^74^.

However, hydrated minerals have also been identified in connection to ancient water-rich settings at Meridiani Planum^75^, Gusev crater^75^, and Gale crater^76,77^. Hence, we consider the alternative that clods formed by freeze-thaw and hydration-dehydration of soluble seawater salts transported onshore by the megatsunami.

The presence of the V1LD's lobate front implies a history of long-term primary morphologic retention, arguing in favor of relatively minor post-emplacement compositional modifications. The deposit's deeper zones would have been more likely to retain seawater salts and megatsunami marine bedload, given their relatively low exposure to impact gardening, aeolian activity, and possible freeze-thaw cycles during high-obliquity phases.

Consequently, the V1L soil salt compositions^72^ could hold information connected to Mars' Late Hesperian northern ocean. For example, cold brine chemistry modeling work of such salt assemblages suggests that the ocean was likely a Mg–Na–Ca–Fe^2+^–SO_4_–Cl–CO_3_–Br-rich brine^75,78^. This solution type is unlike Earth's seawater but like some hypersaline continental brines^79,80^. Their sulfate saturation at ocean temperatures between the mid-260s and low-270s K^75,80^ could have potentially supported life. For example, on Earth, some hypersaline brines with halotolerant taxa are known^81,82^.

Furthermore, as the brine cooled and ice and salts precipitated, the dregs could have remained liquid to lower temperatures by fractionating to a chloride solution, e.g., ~ 252 K for a Na–Cl brine^78^ or even colder perchlorate solutions^83^, perhaps enabling the long-term retention of an ice-covered ocean. While the progressively colder and more hypersaline conditions might have been hostile to life, freeze-dried minerals might still preserve biological signatures. For example, viable halobacteria have been detected within completely dried brine residues after their rehydration and within brines melted after freezing at 203 K^84^.

Our model invoking the possibility of a preserved paleo-oceanic chemistry at the V1L site is not necessarily directly relevant to a controversial updated interpretation suggesting that the V1L-sampled soils contained viable life^85^. Still, it supports eventual reconsideration of those results by further in-situ exploration, given the implication that the regional geology likely was connected to early Mars habitable environments.

#### Pohl crater: submarine hydrothermalism, wet-based, coastal glaciation and mud volcanism

The Late Hesperian ocean may have been sourced from much older, probably Noachian, highland aquifers through the outflow channels^3,4,18,19,53,86–90^. If such aquifers developed during early Mars, when the planet's habitable environments were presumably widespread, their expelled, constituent materials comprise important astrobiological targets. Therefore, we consider that the northern ocean sediments potentially could bear biosignatures, particularly if habitable conditions persisted long-term.

The formation of Pohl as a marine crater would have provided long-term hydrothermal heat, perhaps reproducing aquifer conditions suitable for thermophiles. Abramov and Kring^91^ numerically simulated the post-impact cooling for craters 100 km in diameter formed in submerged targets (Pohl is 110 km). Their simulations indicate that the impact-dispersed heat could have sustained hydrothermal activity for ~ 300,000 years. In connection to Pohl's regional stratigraphy, our results suggest that marine sediments, mostly consisting of submerged portions of the older megatsunami, covered the crater and its surrounding (Fig. 8e). Subsequently, these deposits were largely buried by the younger megatsunami as it overran Pohl with materials expelled long after the ocean formed, also suggesting that at that time voluminous mud-rich strata likely covered the ocean floor (Fig. 8e). Hence, Pohl and its periphery might include marine strata of diverse ages, potentially bearing information on how the ocean's habitability and possible life evolved.

Mud volcanoes over these materials could include cryogenic evaporitic or freeze-driven chemical precipitates, some perhaps containing pristine fluid inclusions of frozen seawater. These materials could yield specific information on the ocean's composition and temperature as well as include potential chemical or physical biosignatures.

## Conclusions

The circum-Chryse outflow channels are considered to be the primary source of Mars' Late Hesperian northern ocean^17–19^. These outflow channels converged into the Chryse Planitia northern lowland region^10^, where they comprise the most extensive contiguous catastrophic flooding landscape on the planet (Fig. 2a).

Our paleophysiographic reconstructions indicate that the Chryse Planitia outflow channel floors occur beneath the previously hypothesized higher paleoshoreline stand at − 3800 m (Fig. 2a). Therefore, these terrains comprise the only catastrophic flood geologic record on Mars that was likely submerged beneath the Late Hesperian northern ocean. Furthermore, the younger megatsunami deposit covers most of these outflow channel sections; hence, the area provides a unique stratigraphic framework consisting of marine sedimentation over ocean-generating flood features.

We identified an impact crater, Pohl, which superposes the outflow channel floors and underlies the younger megatsunami deposit (Fig. 2), characterizing it as a highly probable marine crater. Our numerical simulations indicate that Pohl produced a megatsunami that extensively inundated the Chryse Planitia HLB plains. The modeled megatsunami upper reaches closely match the older megatsunami margins as mapped by Rodriguez, et al.^6^, supporting the simulation's accuracy (Fig. 5). These simulations predict that the megatsunami reached the Viking 1 Lander site, thereby explaining the presence of regional run-up overflow indicators. These include (1) a highland-facing lobe on which the lander sits and (2) scour marks on an adjoining wrinkle ridge that extend over an elongated deposit forming part of the V1LD upper reaches. In addition, the run-up erosional patterns also include scour marks clearly recognizable in high-resolution (decameter-scale) views (Fig. 7). The simulations also suggest that the megatsunami backwash could have produced the boulder imbrication observed at the Mars Pathfinder lander site (Fig. 5).

Our paleophysiographic reconstructions suggest that the Chicxulub impact is an Earth analog to Pohl. It also (1) occurred within a shallow marine environment (~ 200 m)^92^, (2) had a transient cavity diameter of ~ 100 km^93^, similar to Pohl, and (3) produced a similar simulated megatsunami (~ 200 m onshore height, inland surges of ~ 300 km with elevation gains of ~ 150 m)^92^.

Our mapping results also carry important paleoenvironmental implications connected to the modification history of the megatsunami record in Chryse Planitia. For example, we find that Pohl's knobby rim is part of a vast field of knobs and mesas scattered throughout the − 3800 m to − 4100 m regression areas of Chryse Planitia (Fig. 3). Some knobs superpose outflow channel streamlined islands (Fig. 3), and many occupy terrains outside the outflow channels, indicating that the field's formation postdated outflow channel activity. At some locations, the field includes apparently glaciated terrains (Fig. 3), suggesting that a previously proposed circum-oceanic glacial erosion^6,63,67,68^ contributed to the field's origin. Furthermore, the presence of possible tunnel channels and eskers superposed on the younger megatsunami deposit (e.g., Fig. S3d) would indicate that the glaciation phase persisted after the ocean's coastal regression.

Another form of megatsunami resurfacing concerns the high abundance of previously proposed mud volcanoes^39^ over the younger megatsunami deposit (e.g., Fig. S3e) (proposed to mostly consist of waves that froze during run-up). We consider a causative association in which the gradual release of entrapped seawater could have facilitated the eruptions (Fig. 8f).

## Supplementary Information

Supplementary Video 1.

Supplementary Video 2.

Supplementary Video 3.

Supplementary Video 4.

Supplementary Video 5.

Supplementary Video 6.

Supplementary Information 1.

Supplementary Information 2.

Supplementary Information 3.

## References

[1] Milton DJ (1973). Water and processes of degradation in the Martian landscape. J. Geophys. Res..

[2] Masursky H (1973). An overview of geological results from Mariner 9. J. Geophys. Res..

[3] Baker VR, Milton DJ (1974). Erosion by catastrophic floods on Mars and Earth. Icarus.

[4] Sharp RP, Malin MC (1975). Channels on Mars. GSA Bull..

[5] Masursky H., Crabill N. L. (1976). The Viking Landing Sites: Selection and Certification. Science.

[6] Rodriguez JAP (2016). Tsunami waves extensively resurfaced the shorelines of an early Martian ocean. Sci. Rep..

[7] Christensen, P. R., Gorelick, N. S., Mehall, G. L. & Murray, K. C. THEMIS Public Data Releases, Planetary Data System node, Arizona State University, http://themis-data.asu.edu. (2006).

[8] Baker VR, Kochel RC (1979). Martian channel morphology: Maja and Kasei Valles. J. Geophys. Res. Solid Earth.

[9] Baker VR (1982). The channels of Mars.

[10] Tanaka, K. L. *et al.* Geologic map of Mars. U.S. Geological Survey Scientific Investigations Map 3292, scale 1:20,000,000, pamphlet 43 p., 10.3133/sim3292 (2014).

[11] Baker VR (2015). Fluvial geomorphology on Earth-like planetary surfaces: A review. Geomorphology.

[12] Binder AB, Arvidson RE, Guinness EA, Jones KL, Morris EC, Mutch TA, Pieri DC, Sagan C (1977). The geology of the Viking Lander 1 site. J. Geophys. Res..

[13] Sharp RP, Malin MC (1984). Surface geology from Viking landers on Mars: A second look. GSA Bull..

[14] Shorthill RW, Hutton RE, Moore HJ, Scott RF, Spitzer CR (1976). The Viking Landing Sites: Selection and Certification. Science.

[15] Parker TJ, Gorsline DS, Saunders RS, Pieri DC, Schneeberger DM (1993). Coastal geomorphology of the Martian northern plains. J. Geophys. Res.: Planets.

[16] Parker TJ, Saunders RS, Schneeberger DM (1989). Transitional morphology in West Deuteronilus Mensae, Mars: Implications for modification of the lowland/upland boundary. Icarus.

[17] Baker VR (1991). Ancient oceans, ice sheets and the hydrological cycle on Mars. Nature.

[18] Clifford SM (1993). A model for the hydrologic and climatic behavior of water on Mars. J. Geophys. Res.: Planets.

[19] Clifford SM, Parker TJ (2001). The Evolution of the Martian Hydrosphere: Implications for the Fate of a Primordial Ocean and the Current State of the Northern Plains. Icarus.

[20] Craddock RA, Crumpler LS, Aubele JC, Zimbelman JR (1997). Geology of central Chryse Planitia and the Viking 1 landing site: Implications for the Mars Pathfinder mission. J. Geophys. Res.: Planets.

[21] Costard F (2017). Modeling tsunami propagation and the emplacement of thumbprint terrain in an early Mars ocean. J. Geophys. Res.: Planets.

[22] Costard F (2019). The Lomonosov Crater Impact Event: A Possible Mega-Tsunami Source on Mars. J. Geophys. Res.: Planets.

[23] De Blasio FV (2020). Frontal Aureole Deposit on Acheron Fossae ridge as evidence for landslide-generated tsunami on Mars. Planet. Space Sci..

[24] Di Pietro I, Séjourné A, Costard F, Ciążela M, Rodriguez JAP (2021). Evidence of mud volcanism due to the rapid compaction of martian tsunami deposits in southeastern Acidalia Planitia, Mars. Icarus.

[25] Ormö J, Dohm JM, Ferris JC, Lepinette A, Fairén AG (2004). Marine-target craters on Mars? An assessment study. Meteorit. Planet. Sci..

[26] Villiers GD, King DT, Marzen LJ (2010). A study of candidate marine target impact craters in Arabia Terra, Mars. Meteoritics Planet. Sci..

[27] Tanaka, K. L., Skinner, J. A. & Hare, T. M. Geologic map of the northern plains of Mars. U.S. Geological Survey Scientific Investigations Map 2888, scale ranges from 1:15,000,000 to 1:7,500,000, pamphlet 27 p., 10.3133/sim2888 (2005).

[28] Kreslavsky MA, Head JW (2002). Fate of outflow channel effluents in the northern lowlands of Mars: The Vastitas Borealis Formation as a sublimation residue from frozen ponded bodies of water. J. Geophys. Res.: Planets.

[29] Frey HV (2006). Impact constraints on, and a chronology for, major events in early Mars history. J. Geophys. Res.: Planets.

[30] Buczkowski DL, Frey HV, Roark JH, McGill GE (2005). Buried impact craters: A topographic analysis of quasi-circular depressions, Utopia Basin, Mars. J. Geophys. Res.: Planets.

[31] Worthington AM, Cole RS (1900). Impact with a liquid surface studied by the aid of instantaneous photography: Paper II. Philosophical Transactions of the Royal Society A.

[32] Malin MC (2007). Context camera investigation on board the mars reconnaissance orbiter. J. Geophys. Res.: Planets.

[33] Dickson, J. L., Kerber, L. A., Fassett, C. I. & Ehlmann, B. L. A global, blended CTX mosaic of Mars with vectorized seam mapping: a new mosaicking pipeline using principles of non-destructive image editing, Lunar Planet. Sci. Conf. 49, abstract 2480. http://murray-lab.caltech.edu/CTX/. (2018).

[34] Christensen PR (2001). Mars Global Surveyor Thermal Emission Spectrometer experiment: Investigation description and surface science results. J. Geophys. Res.: Planets.

[35] Edwards CS (2011). Mosaicking of global planetary image datasets: 1. Techniques and data processing for Thermal Emission Imaging System (THEMIS) multi-spectral data. J. Geophys. Res.: Planets.

[36] Fergason, R. L., Hare, T. M. & Laura, J. HRSC and MOLA Blended Digital Elevation Model at 200m v2. Astrogeology PDS Annex, U.S. Geological Survey. http://bit.ly/HRSC_MOLA_Blend_v0. (2018).

[37] McEwen AS (2007). Mars reconnaissance orbiter's high resolution imaging science experiment (HiRISE). J. Geophys. Res.: Planets.

[38] McNeil JD, Fawdon P, Balme MR, Coe AL (2021). Morphology, Morphometry and Distribution of Isolated Landforms in Southern Chryse Planitia, Mars. J. Geophys. Res.: Planets.

[39] Brož P, Hauber E, van de Burgt I, Špillar V, Michael G (2019). Subsurface Sediment Mobilization in the Southern Chryse Planitia on Mars. J. Geophys. Res.: Planets.

[40] Noble, C. R. *et al. *ALE3D: An Arbitrary Lagrangian-Eulerian Multi-Physics Code. United States. 10.2172/1361589. https://www.osti.gov/servlets/purl/1361589 (2017).

[41] Collins, G. S. *et al.* iSALE-Dellen manual. 10.6084/m9.figshare.3473690.v2 (2016).

[42] Citron RI, Manga M, Hemingway DJ (2018). Timing of oceans on Mars from shoreline deformation. Nature.

[43] Sholes SF, Rivera-Hernández F (2022). Constraints on the uncertainty, timing, and magnitude of potential Mars oceans from topographic deformation models. Icarus.

[44] Duran S, Coulthard TJ, Baynes ERC (2019). Knickpoints in Martian channels indicate past ocean levels. Sci. Rep..

[45] Wordsworth RD (2016). The Climate of Early Mars. Annu. Rev. Earth Planet. Sci..

[46] Turbet M, Forget F, Head JW, Wordsworth R (2017). 3D modelling of the climatic impact of outflow channel formation events on early Mars. Icarus.

[47] Ivanov BA (2001). Mars/moon cratering rate ratio estimates. Space Sci. Rev..

[48] Hauck SA, Phillips RJ (2002). Thermal and crustal evolution of Mars. J. Geophys. Res.: Planets.

[49] Yoshizaki T, McDonough WF (2020). The composition of Mars. Geochim. Cosmochim. Acta.

[50] Ma Y, Williams IP, Ip WH, Chen W (2002). The velocity distribution of periodic comets and the meteor shower on Mars. A&A.

[51] Mandli KT (2016). Clawpack: building an open source ecosystem for solving hyperbolic PDEs. PeerJ Comput. Sci..

[52] Golombek MP (1997). Overview of the mars pathfinder mission and assessment of landing site predictions. Science.

[53] Rodriguez JAP (2019). The 1997 Mars Pathfinder Spacecraft Landing Site: Spillover Deposits from an Early Mars Inland Sea. Sci. Rep..

[54] González, F. G. *et al.* Validation of the GeoClaw Model: NTHMP MMS Tsunami Inundation Model Validation Workshop. (2011).

[55] Mangold N, Allemand P (2001). Topographic analysis of features related to ice on Mars. Geophys. Res. Lett..

[56] Squyres SW (1978). Martian fretted terrain: Flow of erosional debris. Icarus.

[57] Li H, Robinson MS, Jurdy DM (2005). Origin of martian northern hemisphere mid-latitude lobate debris aprons. Icarus.

[58] Chuang FC, Crown DA (2005). Surface characteristics and degradational history of debris aprons in the Tempe Terra/Mareotis fossae region of Mars. Icarus.

[59] Berman DC, Crown DA, Joseph ECS (2015). Formation and mantling ages of lobate debris aprons on Mars: Insights from categorized crater counts. Planet. Space Sci..

[60] Chuang, F. C. & Crown, D. A. Geologic map of MTM 35337, 40337, and 45337 quadrangles, Deuteronilus Mensae region of Mars: U.S. Geological Survey Scientific Investigations Map 3079. (2009).

[61] Pierce TL, Crown DA (2003). Morphologic and topographic analyses of debris aprons in the eastern Hellas region, Mars. Icarus.

[62] Carr MH, Head JW (2003). Oceans on Mars: An assessment of the observational evidence and possible fate. J. Geophys. Res.: Planets.

[63] Kargel JS (1995). Evidence of ancient continental glaciation in the Martian northern plains. J. Geophys. Res.: Planets.

[64] Gallagher C, Balme M (2015). Eskers in a complete, wet-based glacial system in the Phlegra Montes region, Mars. Earth Planet. Sci. Lett..

[65] Banks ME (2009). An analysis of sinuous ridges in the southern Argyre Planitia, Mars using HiRISE and CTX images and MOLA data. J. Geophys. Res.: Planets.

[66] Kargel JS, Strom RG (1992). Ancient glaciation on Mars. Geology.

[67] Fairén AG (2011). Cold glacial oceans would have inhibited phyllosilicate sedimentation on early Mars. Nat. Geosci..

[68] Schmidt F (2022). Circumpolar ocean stability on Mars 3 Gy ago. Proc. Natl. Acad. Sci..

[69] Davila AF (2013). Evidence for Hesperian glaciation along the Martian dichotomy boundary. Geology.

[70] Head JW (2005). Tropical to mid-latitude snow and ice accumulation, flow and glaciation on Mars. Nature.

[71] van Gasselt S, Hauber E, Neukum G (2007). Cold-climate modification of Martian landscapes: A case study of a spatulate debris landform in the Hellas Montes Region, Mars. J. Geophys. Res.: Planets.

[72] Toulmin P, Clark BC, Baird AK, Keil K, Rose HJ (1976). Preliminary Results from the Viking X-ray Fluorescence Experiment: The First Sample from Chryse Planitia, Mars. Science.

[73] Clark BC (1982). Chemical composition of Martian fines. J. Geophys. Res.: Solid Earth.

[74] Keller JM (2006). Equatorial and midlatitude distribution of chlorine measured by Mars Odyssey GRS. J. Geophys. Res.: Planets.

[75] Marion GM, Catling DC, Kargel JS (2009). Br/Cl partitioning in chloride minerals in the Burns formation on Mars. Icarus.

[76] Vaniman DT (2018). Gypsum, bassanite, and anhydrite at Gale crater, Mars. Am. Miner..

[77] Rapin W (2019). An interval of high salinity in ancient Gale crater lake on Mars. Nat. Geosci..

[78] Marion, G. & Kargel, J. S. Cold Aqueous Planetary Geochemistry with FREZCHEM: From Modeling to the Search for Life at the Limits. 1st edn. (Springer Berlin, Heidelberg, 2008). 10.1007/978-3-540-75679-8

[79] Long DT (1992). Formation of alunite, jarosite and hydrous iron oxides in a hypersaline system: Lake Tyrrell, Victoria, Australia. Chem. Geol..

[80] Marion GM, Kargel JS, Catling DC (2008). Modeling ferrous–ferric iron chemistry with application to martian surface geochemistry. Geochim. Cosmochim. Acta.

[81] Sánchez-Andrea I, Rodríguez N, Amils R, Sanz JL (2011). Microbial Diversity in Anaerobic Sediments at Río Tinto, a Naturally Acidic Environment with a High Heavy Metal Content. Appl. Environ. Microbiol..

[82] DasSarma S, DasSarma P, Laye VJ, Schwieterman EW (2020). Extremophilic models for astrobiology: haloarchaeal survival strategies and pigments for remote sensing. Extremophiles.

[83] Toner JD, Catling DC, Light B (2014). The formation of supercooled brines, viscous liquids, and low-temperature perchlorate glasses in aqueous solutions relevant to Mars. Icarus.

[84] Crisler JD, Newville TM, Chen F, Clark BC, Schneegurt MA (2012). Bacterial growth at the high concentrations of magnesium sulfate found in martian soils. Astrobiology.

[85] Levin GV, Straat PA (2016). The case for extant life on mars and its possible detection by the viking labeled release experiment. Astrobiology.

[86] Carr MH (1979). Formation of Martian flood features by release of water from confined aquifers. J. Geophys. Res.: Solid Earth.

[87] Rotto, S. & Tanaka, K. L. Geologic/geomorphologic map of the Chryse Planitia region of Mars. Geologic map of the northern plains of Mars. U.S. Geological Survey Scientific Investigations Map 2441, scale 1:500,000, pamphlet 10 p., 10.3133/i2441 (1995).

[88] Rodriguez JAP (2015). Martian outflow channels: How did their source aquifers form and why did they drain so rapidly?. Sci. Rep..

[89] Scott, D. H. & Carr, M. H. Geologic map of Mars. Geologic map of Mars. U.S. Geological Survey Scientific Investigations Map 1083, scale 1:25,000,000. 10.3133/i1083 (1978).

[90] Scott, D. H. & Tanaka, K. L. Geologic map of the western equatorial region of Mars. Geologic map of Mars. U.S. Geological Survey Scientific Investigations Map1802A, scale 1:15,000,000. 10.3133/i1802A (1986).

[91] Abramov O, Kring DA (2005). Impact-induced hydrothermal activity on early Mars. J. Geophys. Res.: Planets.

[92] Matsui, T., Imamura, F., Tajika, E., Nakano, Y. & Fujisawa, Y. In Catastrophic events and mass extinctions: Impacts and beyond. Vol. **356** (eds. Christian Koeberl & Kenneth G. MacLeod) 69–77 (*Geological Society of America*, 2002). ISBN-10 0813723566

[93] Morgan J (1997). Size and morphology of the Chicxulub impact crater. Nature.

