# Supplementary material for: Evidence of an oceanic impact and megatsunami sedimentation in Chryse Planitia, Mars
Source: Sci Rep. 2022 Dec 1;12:19589. doi: 10.1038/s41598-022-18082-2 (PMC9715952; doi:10.1038/s41598-022-18082-2)
Supplement: Supplementary file 7 — Supplementary Information 1. [file 41598_2022_18082_MOESM7_ESM.docx]

**Supplementary materials to:**

**Evidence of an Oceanic Impact and Megatsunami Sedimentation in Chryse Planitia, Mars**

J. Alexis P. Rodriguez^1^*, Darrel K. Robertson^2^, Jeffrey S. Kargel^1^, Victor R. Baker^3^, Daniel C. Berman^1^, Jacob Cohen^2^, Francois Costard^4^, Goro Komatsu^5^, Anthony Lopez^1^, Hideaki Miyamoto^6^, Mario Zarroca^7^ (alphabetical listing after 3^rd^ author, see contributing paragraph for details).

*^1^Planetary Science Institute, 1700 East Fort Lowell Road, Suite 106, Tucson, AZ 85719-2395, USA.*

*^2^NASA Ames Research Center, Moffett Field, CA 94035, USA.*

*^3^Department of Hydrology & Atmospheric Sciences, University of Arizona, Tucson, AZ 85721, USA.*

*^4^GEOPS-Géosciences Paris Sud, Université Paris-Sud, CNRS, Université Paris-Saclay, 91405 Orsay, France.*

*^5^International Research School of Planetary Sciences, Università D'Annunzio, Viale Pindaro 42, 65127 Pescara, Italy.*

*^6^Department of Systems Innovation, University of Tokyo, Tokyo 113-8656, Japan.*

*^7^External Geodynamics and Hydrogeology Group, Department of Geology, Autonomous University of Barcelona, 08193 Bellaterra, Barcelona, Spain.*

Corresponding author: Alexis Rodriguez ([alexis@psi.edu](mailto:alexis@psi.edu)/[alexis1709@gmail.com](mailto:alexis1709@gmail.com))

**(1) Additional Details on Impact-tsunami Generation Numerical Modeling Methodology: Weak vs. Strong Ground**

For the two ground strength models, the weaker model, adapted from Ai and Ahrens ^1^, used a formulation from Johnson, et al. ^2^, with elastic modulus 0.2 Mbar, Poisson ratio 0.25, tensile failure pressure 13 MPa, uniaxial tensile failure stress 10.5 MPa, shear failure stress 19.8 MPa, uniaxial compressive failure stress of 160 MPa, maximum failure stress 2730 MPa and a strain rate coefficient of 0.05. The rock was assumed to be brittle and failed rock was assumed to have zero shear failure stress, coefficient of friction at zero pressure of 1.435 (same as intact rock) and maximum failure stress of 500 MPa.

The stronger rock model used that of Collins, et al. ^3^. This model has a Poisson ratio of 0.3 and has weaker tensile pressure of 5 MPa, shear failure of 10 MPa, but has a coefficient of friction of 2. Being larger than √3=1.73 means there is no uniaxial compressive failure until the maximum failure stress of 2500 MPa is reached. Failed rock has a shear strength of 0.01 MPa and a coefficient of friction of 0.6.


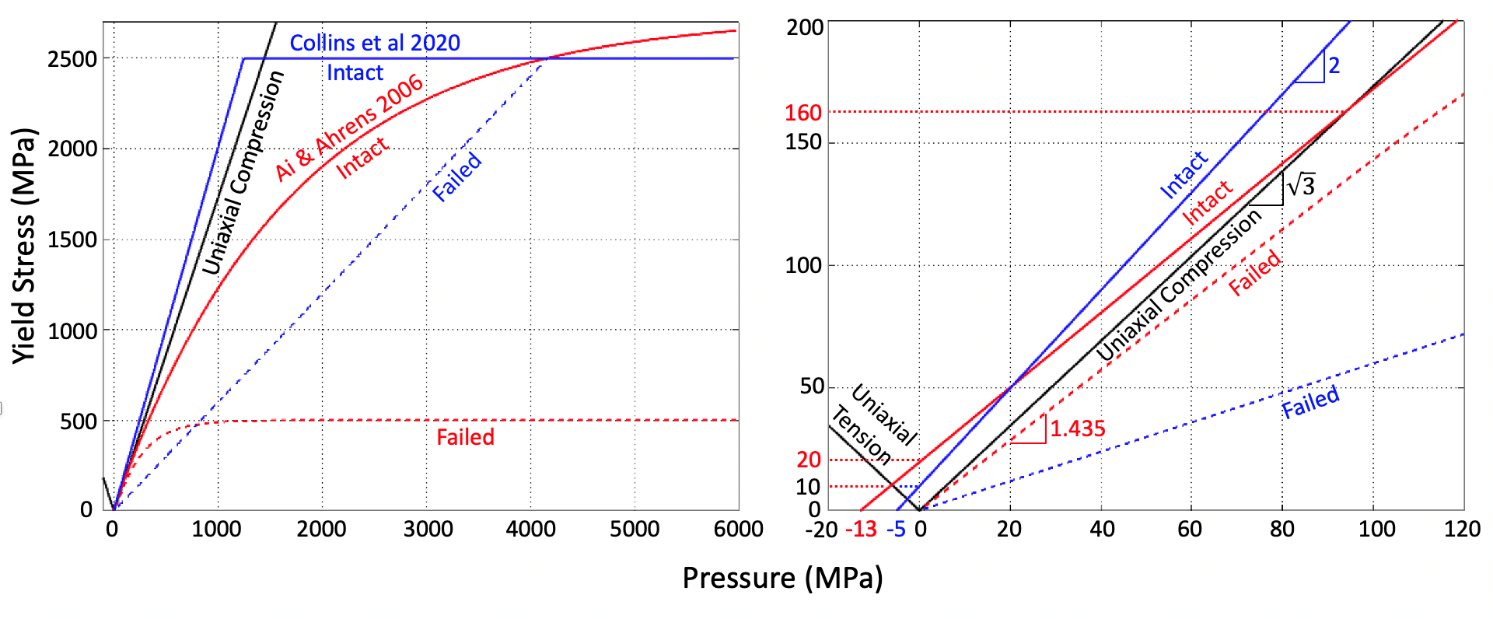


*Ground strength models used in the simulations. The "weaker" model was adapted from Ai and Ahrens ^1^. When it failed, the shear yield stress dropped to zero. The "stronger" model is from Collins, et al. ^3^ and has a residual shear yield stress of 0.01 MPa.*

**(2) Supplementary Figures**

**
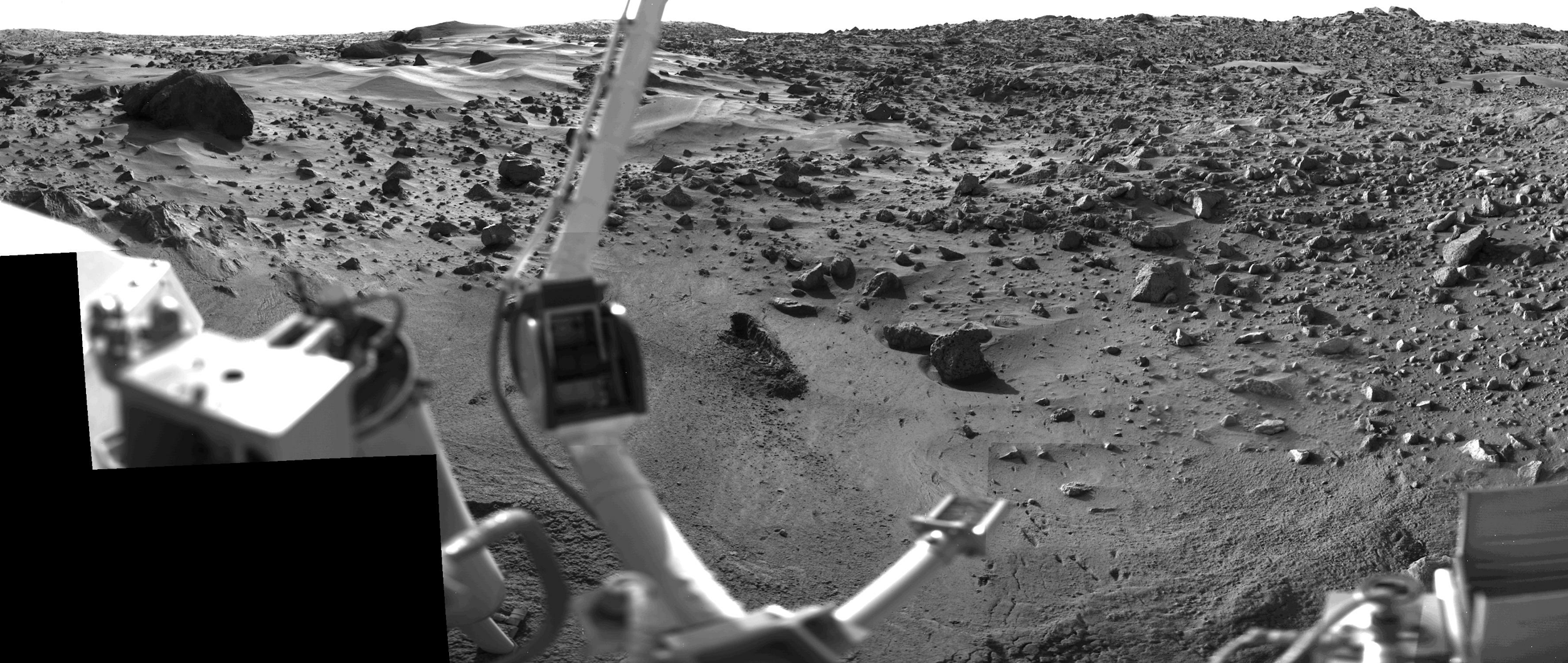
**

**Figure S1** View of the V1L site showing a boulder-rich surface, locally covered by aeolian mantles. Image source: <https://photojournal.jpl.nasa.gov/jpeg/PIA03163.jpg>. Credit to NASA.

**
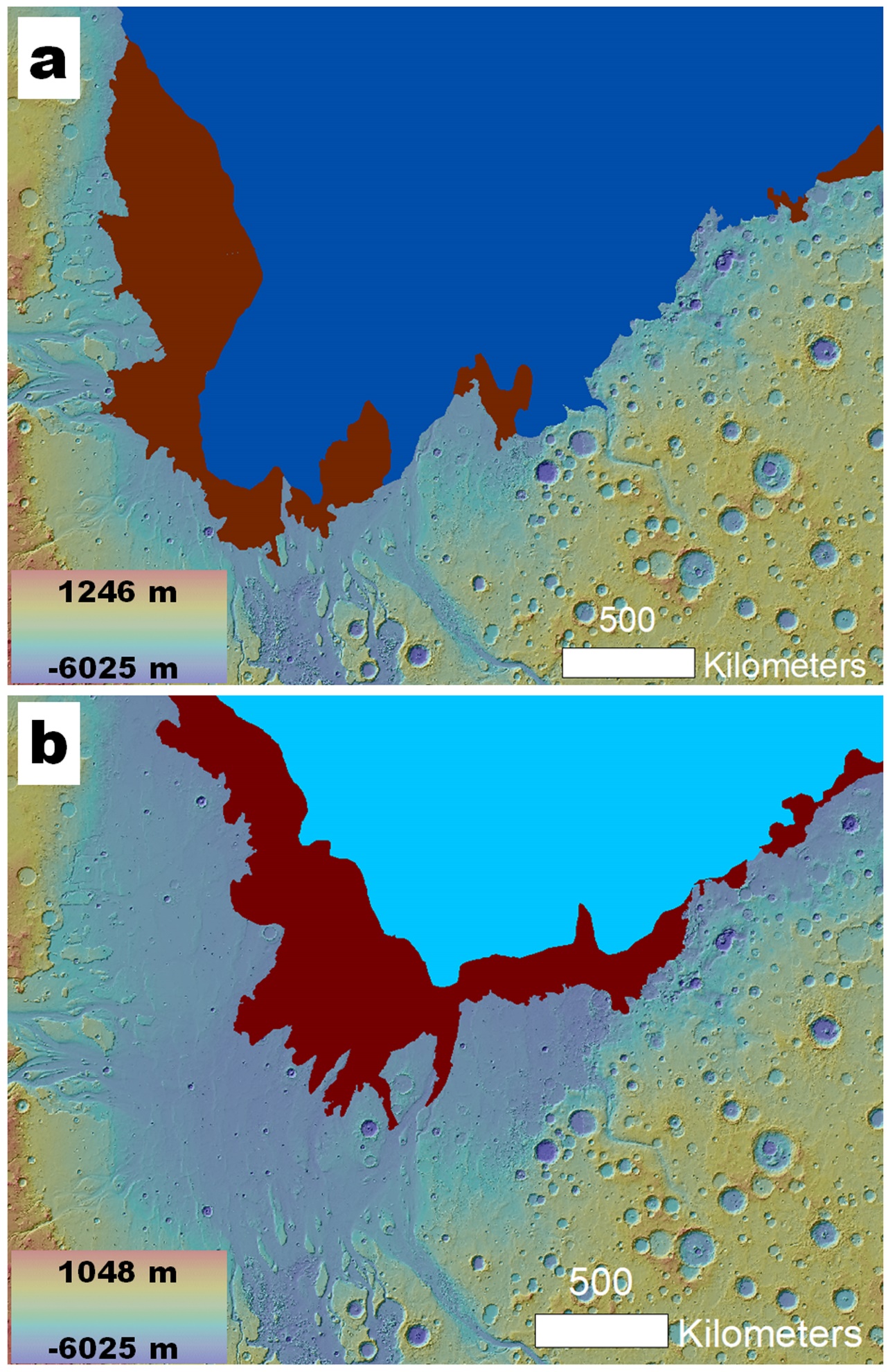
**

**Figure S2 (a)** View of Chryse Planitia, including part of the northern ocean (dark blue) with a paleoshoreline at -3,800 m. The brown lobes depict the extent of the older megatsunami. **(b)** View of Chryse Planitia, including part of the northern ocean (light blue) with a paleoshoreline at -4,100 m. The red lobes depict the extent of the younger megatsunami. The reconstructions are based on the study performed by Rodriguez, et al. ^4^. Both panels use MOLA DEM bases (460 m/pixel, credit: MOLA Science Team, MSS, JPL, NASA). We produced this figure using Esri's ArcGIS 10.3 (<http://www.esri.com/software/arcgis>).


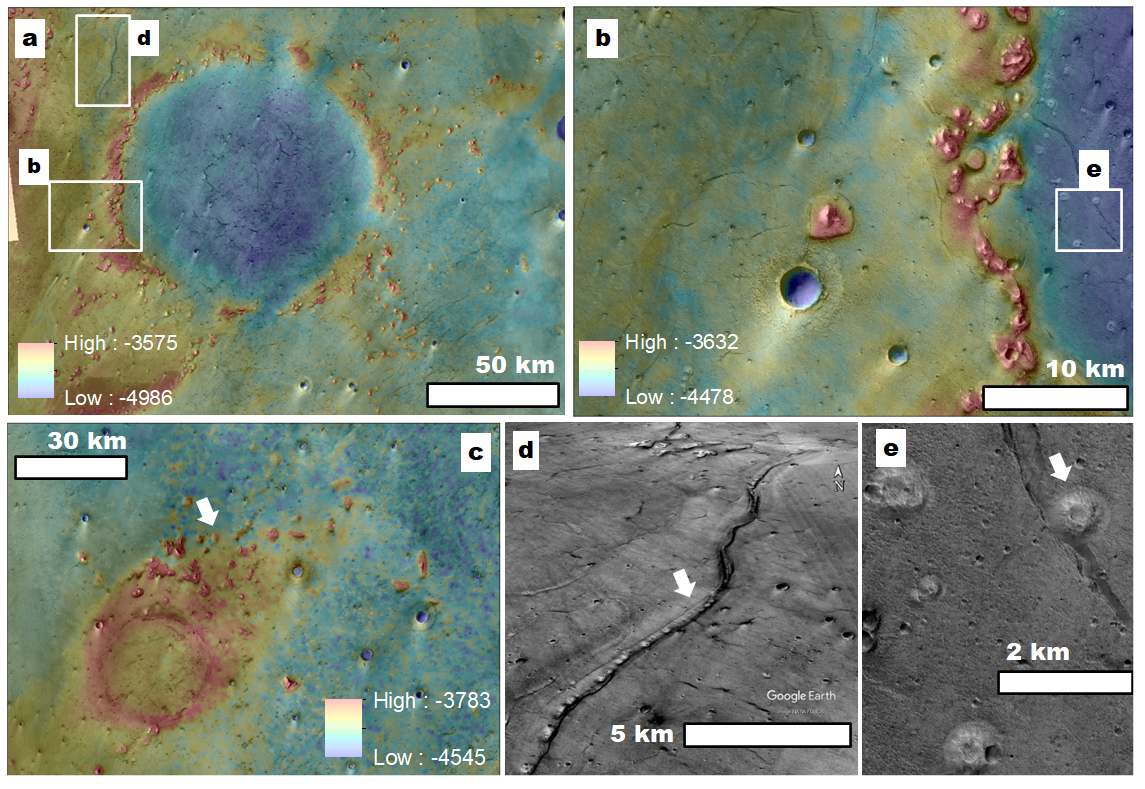
 **Figure S3 (a)** View of Pohl crater showing its knobby rim and surrounding plains. **(b)** Close-up view of Pohl's western rim area showing multiple knobs without flow "tails". The panel **(e)** inset identifies a cluster of possible mud volcanoes. **(c)** View of streamlined island superposed by knobs (also identified by red arrow in Fig. 2b). **(d)** View of possible esker ~15 km northwest of Pohl (white arrow). **(e)** Close-up view of possible mud volcanoes (e.g., white arrow) occupying Pohl's interior plains consisting of overflow younger megatsunami deposits. The noted mud volcano candidate has a flank that partly covers a trough segment, pointing to a formation that could have significantly postdated emplacement. **(a-c)** Color-coded shaded-relief MOLA digital elevation model (460 m/pixel, credit: MOLA Science Team, MSS, JPL, NASA) over part of a CTX mosaic (6 m/pixel, credit: NASA/JPL/Malin Space Science Systems (<https://www.msss.com/mro/marci/images/tips/mediatips.html>). **(d)** Perspective CTX view. Credits to Google. **(e)** Park of a CTX mosaic (6 m/pixel, credit: NASA/JPL/Malin Space Science Systems (<https://www.msss.com/mro/marci/images/tips/mediatips.html>). We produced this figure using Esri's ArcGIS 10.3 (<http://www.esri.com/software/arcgis>).

**
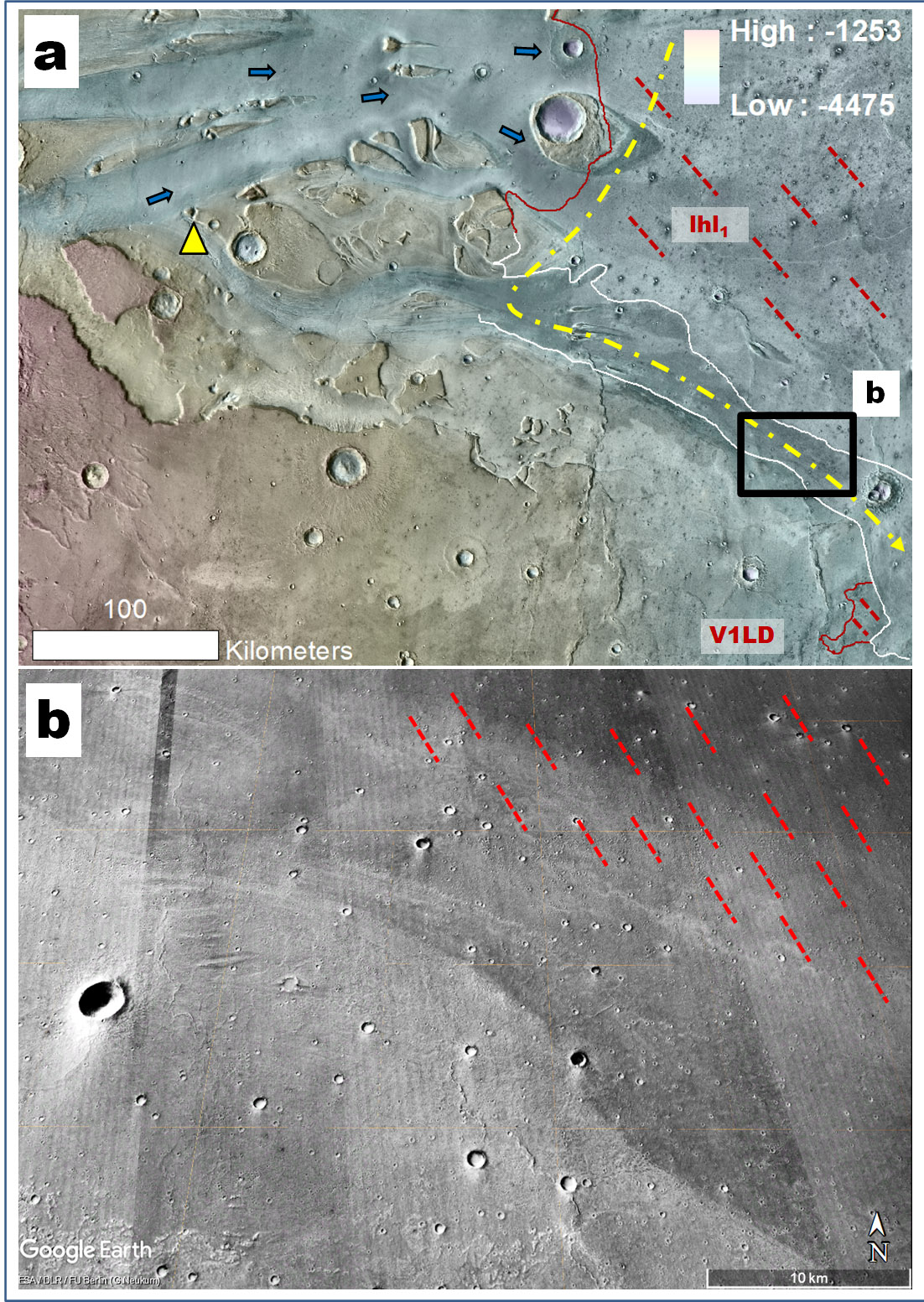
**

**Figure S4 (a)** View of the lowest reaches of Kasei Valles (blue arrows), including the location of the V1LD. The dashed red lines indicate areas covered by the older megatsunami (unit lHl_1_). The white lines outline the margins of a channel cutting into the deposit. The channel is a higher branch (hence older) extending from a wider and deeper section of the outflow channel (yellow triangle). The deeper section of Kasei Valles, however, has downstream reaches intruded by a section of the older megatsunami. These megatsunami materials lack evidence of overflow and dissection (continuous red line), indicating that the younger flooding (responsible for the older megatsunami deposit's dissection) was limited to the older channel (yellow triangle). The dashed yellow line shows the backwash motion at this location, as indicated in our simulation in Figure S5. Color MOLA DEM (460 m/pixel, credit: MOLA Science Team, MSS, JPL, NASA) over a THEMIS nighttime IR global layer (<http://www.mars.asu.edu/data/>, 100 m/pixel, credit: Christensen, et al. ^5^). **(b)** Close-up CTX view of the channel cut into the older megatsunami deposit. The orange lines mark the megatsunami deposit's erosional margin. We produced this figure using Esri's ArcGIS 10.3 (<http://www.esri.com/software/arcgis>).

**
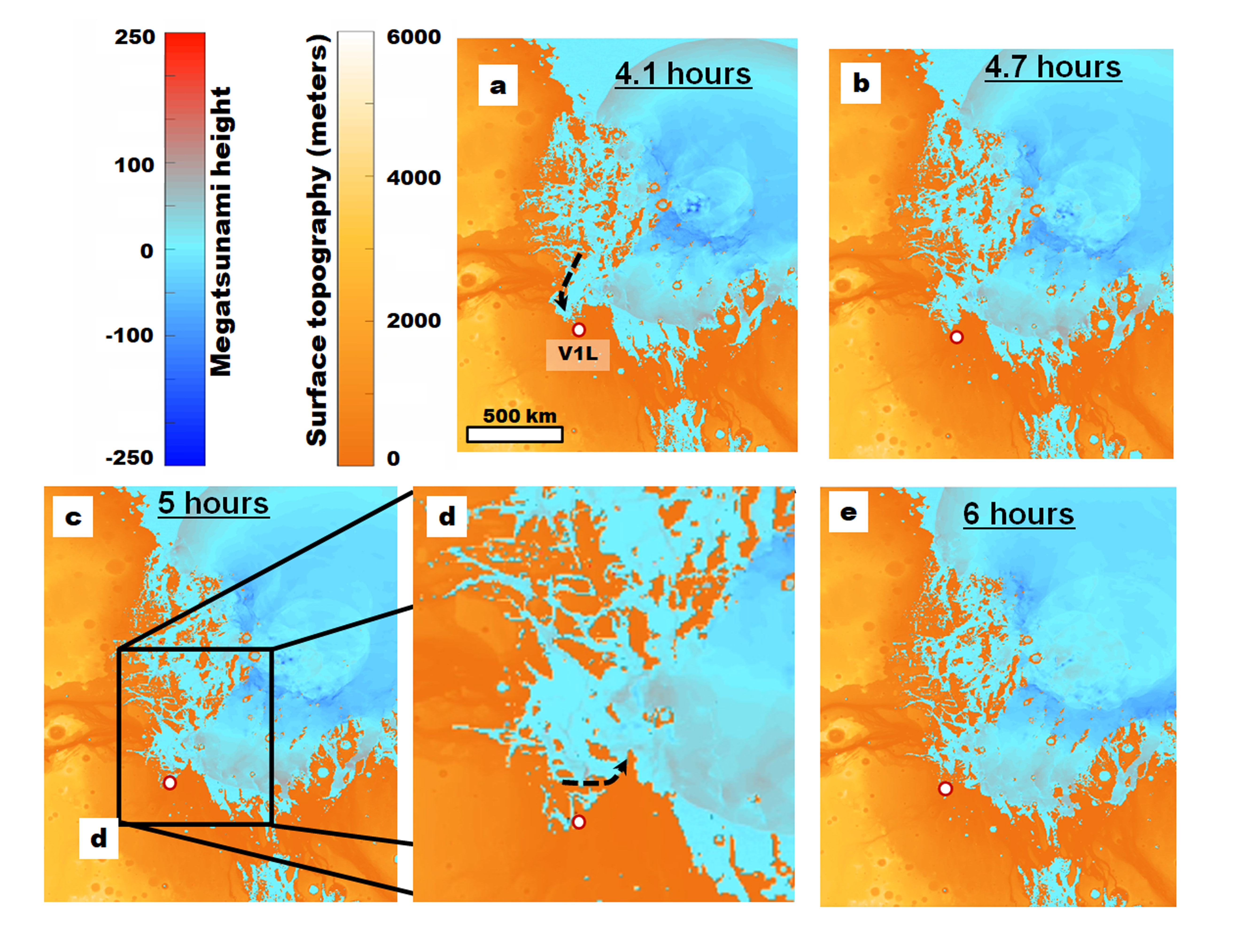
Figure S5** Frames from our megatsunami weak ground simulation. **(a, b)** The megatsunami reaches the V1L site between hours 4.1 and 4.7. **(c)** At hour 5, the megatsunami's front in the region shifts direction towards the northern plains dissecting the channel shown in Figs. 1b and S4. **(d)** During the following hour, the wave backwashes into the ocean. The black arrows in panels **(a)** and **(d)** illustrate the overall trajectory. The megatsunami height is in meters. The topographic ranges in the DEMs represent the regional relief with a zero-meter base.

**
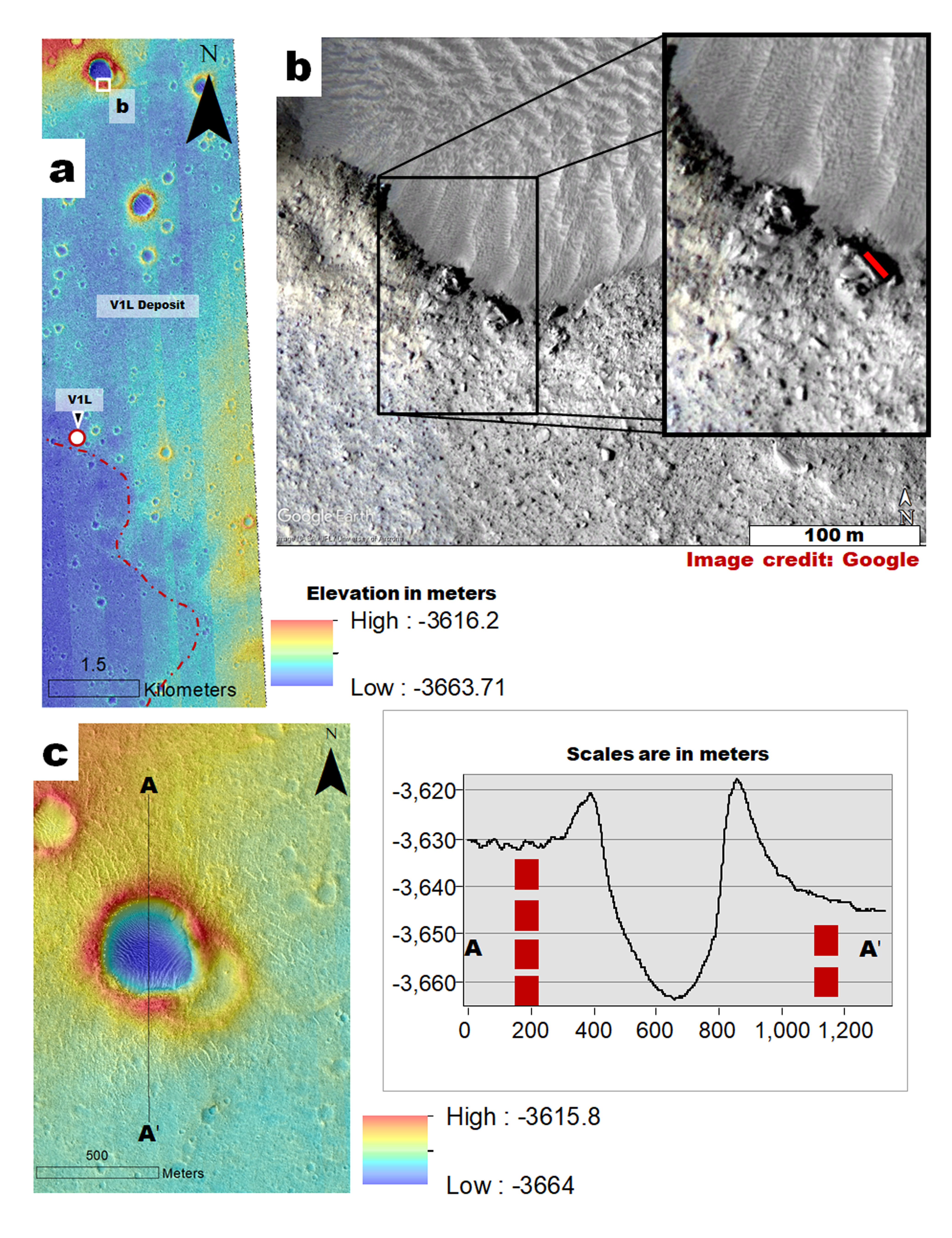
**

**Figure S6** Observations supporting that the depositional lobe on which the V1L site is located is a boulder-rich deposit. **(a)** View of the V1L lobe's upper reaches centered at 22°16' N, 47°56' W (red-circled white dot identifies the landing site). The dashed red line traces part of the lobe's front (context in Figs. 1b and 7a, b). The image is a transparent DTM generated from stereo pair PSP_001719_2025 and PSP_001521_2025 blended over image PSP_001719_2025 (~25 pixels/degree, credit: NASA/JPL/University of Arizona (<https://www.uahirise.org/media/usage.php>). **(b)** Close-up view of the V1L deposit's boulder-rich stratigraphy exposed along the southern margin of a ~450-m-in-diameter impact crater that is located ~6 km north of the landing site (location in panel **(a)**). The red line traces a boulder ~ 13 m across (image PSP_001719_2025, ~25 pixel/degree, credit: NASA/JPL/University of Arizona (<https://www.uahirise.org/media/usage.php>)).  **(c)** Topographic view of the crater partly shown in panel **(b)**. The red squares within the elevation profile trace the approximate thickness of the boulder-rich deposit measured as a function of the crater excavation depth below its adjoining plains (~30 m). The image is a transparent digital terrain model generated from the stereo pair PSP_001719_2025 and PSP_001521_2025 blended over image PSP_001719_2025 (~25 pixels/degree, credit: NASA/JPL/University of Arizona (<https://www.uahirise.org/media/usage.php>)).

**
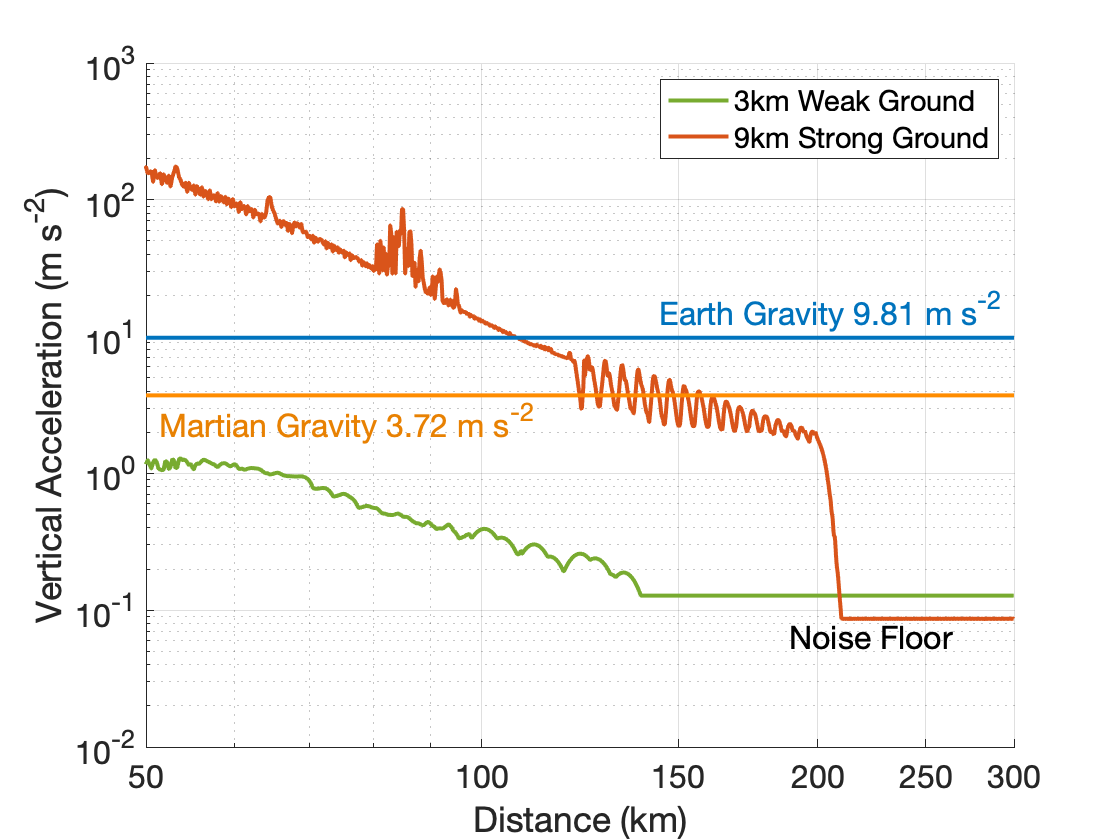
**

**Figure S7** Seismic waves from the impacts of 3 and 9-km diameter asteroids into weak and strong ground, respectively. The smaller impact generates seismic waves with vertical accelerations less than Martian gravity everywhere outside the crater rim at 55 km. However, the vertical acceleration of seismic waves generated by the larger impact exceeds Martian gravity out to over 150 km from the impact point. This seismicity could have injected voluminous seafloor materials into the megatsunami. The noise floor is the simulation left over from the initial equilibrium calculation. At later times, there is a stronger seismic wave caused by the reflection from the floor of the simulation 100 km deep, but this is not realistic. Obtaining reliable seismic data at longer times would require modeling an even deeper wedge of Mars and accounting for the crust-mantle transition at ~120 km depth and other potential discontinuities ^6^.

**
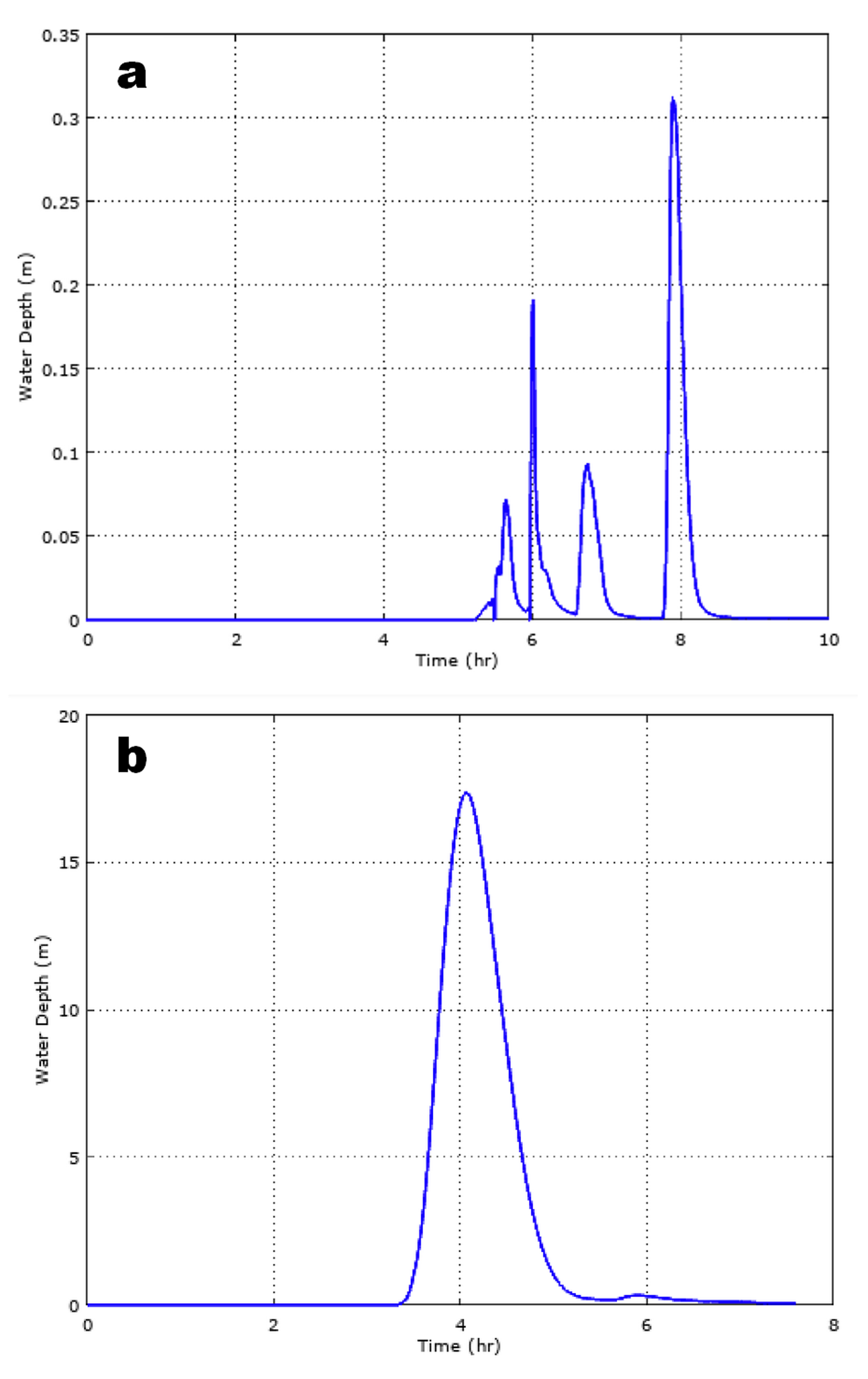
**

**Figure S8** Gauge data at the V1L site for the weak ground (WG) **(a)** and strong ground (SG) simulations **(b)**. **(a)** Several megatsunamis wash over the landing site as waves arrive from different directions, the largest being 0.3m deep. **(b)** A major megatsunami reaches the landing site with a front ~15m deep. The values in both panels, while generalized regional approximations, indicate that regional megatsunami backwash might have started from shallowly inundated depths. Consequential slow backwash is unlikely to have formed channels.

**
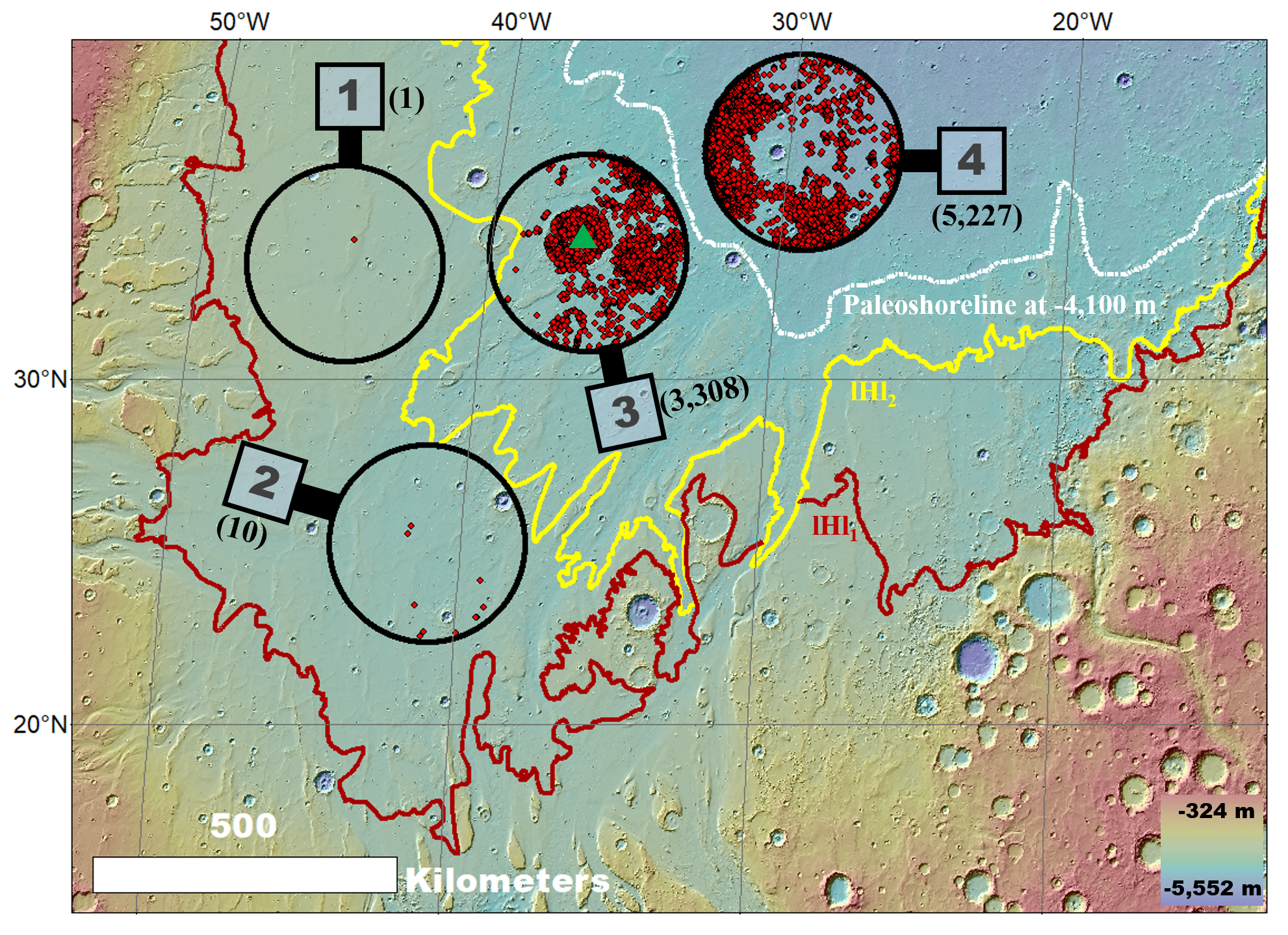
**

**Figure S9** We carried four surveys of possible mud volcano clusters within four circular areas ~300 km in diameter: (1 & 2) older megatsunami (lHl_1_) deposit sampled areas; (3) younger megatsunami (lHl_2_) deposit sampled area (green triangle positioned on Pohl); (4) sampled terrain below the lower paleoshoreline interpreted as part of the frozen ocean residue (~-4,100 m, see Fig. 2a for outflow channel bedform termination at this proposed paleoshoreline). Most mapped possible mud volcanoes consist of pitted cones, generally recognizable in CTX as having comparatively higher surface albedos. Shallow moats surround many of them, and some connect to lobate flow features. We also counted partially degraded (but still recognizable) occurrences of mud volcanoes. While individual points identify single mud volcanoes, this figure's purpose is to quantitatively demonstrate that they are highly abundant over the younger megatsunami deposit and the proposed ocean's frozen residue but are largely absent within the older megatsunami surface. To show the high abundance, we mapped most (but not all) occurrences. Counts for zones 1-4 are, respectively, (1), (10), (3,308) & (5,227). Not all the points are visible due to overlapping within the clusters. Within the younger megatsunami and ocean's frozen residue, some gaps correspond to areas of ejecta deposition or enhanced degradation rates. However, further work is needed to assess the distribution patterns more adequately. Our findings that mud volcanoes cluster largely within the younger megatsunami and frozen ocean residue suggest a connection between seawater retention and mud volcanism in these regions. The mud volcanoes were mapped using a CTX-base (6 m per pixel, credit: NASA/JPL/Malin Space Science Systems (<https://www.msss.com/mro/marci/images/tips/mediatips.html>). The figure base is a Color MOLA DEM (460 m per pixel, credit: MOLA Science Team, MSS, JPL, NASA) over a THEMIS nighttime IR global layer (<http://www.mars.asu.edu/data/>, 100 m per pixel, credit: Christensen, et al. ^5^). We produced this figure using Esri's ArcGIS 10.3 (<http://www.esri.com/software/arcgis>).

**References**

1 Ai, H. A. & Ahrens, T. J. Simulation of dynamic response of granite: A numerical approach of shock-induced damage beneath impact craters. *International Journal of Impact Engineering* **33**, 1-10, doi:<https://doi.org/10.1016/j.ijimpeng.2006.09.046> (2006).

2 Johnson, G. R., Holmquist, T. J. & Beissel, S. R. Response of aluminum nitride (including a phase change) to large strains, high strain rates, and high pressures. *Journal of Applied Physics* **94**, 1639-1646, doi:10.1063/1.1589177 (2003).

3 Collins, G. S. *et al.* A steeply-inclined trajectory for the Chicxulub impact. *Nature Communications* **11**, 1480, doi:10.1038/s41467-020-15269-x (2020).

4 Rodriguez, J. A. P. *et al.* Tsunami waves extensively resurfaced the shorelines of an early Martian ocean. *Scientific Reports* **6**, 25106, doi:10.1038/srep25106 (2016).

5 Christensen, P. R., Gorelick, N. S., Mehall, G. L. & Murray, K. C. THEMIS Public Data Releases, Planetary Data System node, Arizona State University, <<http://themis-data.asu.edu>>. (2006).

6 Khan, A. *et al.* Upper mantle structure of Mars from InSight seismic data. *Science* **373**, 434-438, doi:doi:10.1126/science.abf2966 (2021).
